# The grapevine ABC transporter B family member 15 is a *trans*-resveratrol transporter out of grapevine cells

**DOI:** 10.3389/fpls.2024.1450638

**Published:** 2025-01-20

**Authors:** Ascensión Martínez-Márquez, Viviana Martins, Susana Sellés-Marchart, Hernâni Gerós, Purificación Corchete, Roque Bru-Martínez

**Affiliations:** ^1^ Plant Proteomics and Functional Genomics Group, Department of Biochemistry and Molecular Biology and Soil Science and Agricultural Chemistry, Faculty of Science, University of Alicante, Alicante, Spain; ^2^ Alicante Institute for Health and Biomedical Research (ISABIAL), Alicante, Spain; ^3^ Centre of Molecular and Environmental Biology, Department of Biology, University of Minho, de Gualtar, Braga, Portugal; ^4^ Research Technical Facility, Proteomics and Genomics Division, University of Alicante, Alicante, Spain; ^5^ Department of Plant Physiology, Miguel de Unamuno, University of Salamanca, Salamanca, Spain; ^6^ Multidisciplinary Institute for the Study of the Environment (IMEM), University of Alicante, Alicante, Spain

**Keywords:** ATP binding cassette (ABC) transporter B family member 15, label-free proteomics analysis, grapevine cell culture, resveratrol, transformation, transport, *Vitis vinifera*

## Abstract

Stilbenes, particularly *trans*-resveratrol, play a highly relevant defense role in grapevines as phytoalexin is induced in response to stress. Metabolism and transport of stilbenes can be conveniently investigated in grapevine cell culture since large amounts of *trans*-resveratrol are accumulated in the extracellular medium upon treatment with the elicitor methylated cyclodextrin, either alone or combined with methyl jasmonate. A proteomic approach on grapevine cell membrane fractions was performed to find *trans*-resveratrol transporter candidates. The candidate VvABCB15 was functionally characterized. Its stable expression in both yeast and *Silybum marianum* cells’ heterologous systems led to increased *trans*-resveratrol transport in these hosts. Transient expression in *Vitis* cells showed an enhanced absorbent- or elicitor-assisted accumulation of extracellular *trans*-resveratrol in VvABCB15-expressing or VvGSTU10/VvABCB15-co-expressing cell suspension cultures. Experiments of transient expression in *Vitis* cell suspensions using light-switchable stilbene synthase (pHYH::VvSTS3) and VvABCB15 further confirmed the candidate’s role as a *trans*-resveratrol transporter. VvABCB15-YFP fusion proteins in *Nicotiana* leaf showed localization in the plasma membrane, consistent with a functional role in *trans*-resveratrol transport. This is the first report to provide evidence for the involvement of an ABC transporter B type, VvABCB15, in *trans*-resveratrol transport to the extracellular medium of grapevine cells.

## Introduction

Both abiotic and biotic stresses permanently challenge plants, and to cope with them, plants endow a complex defense and resistance network of constitutive and inducible mechanisms, including phytoalexins. Stilbenes are the phytoalexin group found in grapevines (*Vitis vinifera*) (Langcake and Pryce, 1977), Vitaceae, and few other plant families (Morales et al., 2000). The biosynthetic route leading to stilbenes is a small branch of the general phenylpropanoid pathway consisting of the condensation of the precursor p-coumaroyl-CoA with 3 units of malonyl-CoA catalyzed by stilbene synthase (STS) (Langcake and Pryce, 1977) to produce *trans*-resveratrol (3,4′,5-trihydroxystilbene, *t*-R), which is the most abundant stilbene both in grapevine vegetative tissues and berries and in cultured cells in response to abiotic and biotic stress (Cantos et al., 2003; Wang et al., 2010). This primary stilbene has phytoalexin activity by itself. Still, it can be further metabolized to be converted into more active stilbene phytoalexins such as the methylated derivative pterostilbene (Adrian et al., 1997) by grapevine resveratrol O-methyltransferase (Schmidlin et al., 2008). Furthermore, fungal laccases (Pezet, 1998; Schouten et al., 2002) can generate the dimer ϵ−Viniferin and higher oligomers (Langcake, 1981; Gabaston et al., 2017) having more potent antifungal activity than *t*-R, thus emphasizing the relevance of the extracellular *t*-R metabolism in its role as phytoalexin.

In addition to functioning as phytoalexins, a strong focus on stilbene bioproduction research has been implemented in the last decade (Jeandet et al., 2021) due to a large and diverse number of biological activities attributed mainly to *t*-R, including inhibiting the progression of cardiovascular, carcinogenic, and neurodegenerative diseases as well as the aging process, as confirmed by several *in vitro* assays (Kukreja et al., 2014).

Treatment of grapevine cell culture with either abiotic or biotic elicitors promotes the expression of STS genes and proteins followed by the accumulation of intra- and extracellular resveratrol (Tassoni et al., 2005; Lijavetzky et al., 2008; Martinez-Esteso et al., 2009; Almagro et al., 2014). The extracellular accumulation is particularly abundant when methylated cyclodextrins (MBCDs), alone (Bru et al., 2006) or combined with the phytohormone methyl jasmonate (MeJA), are used as elicitors (Lijavetzky et al., 2008; Martinez-Esteso et al., 2009, 2011; Belchí-Navarro et al., 2012, 2013; Almagro et al., 2014) due to the combination of the strong induction of biosynthetic genes of shikimate, phenylpropanoid, and stilbenoid pathways (Lijavetzky et al., 2008; Almagro et al., 2014) and the ability of cyclodextrins to form inclusion complexes with *t*-R in the extracellular medium (Morales et al., 1998) that protect it from degradation (Jeandet et al., 2021).

Besides biosynthetic genes, a tau-class GST (XM-002275302.2 PREDICTED*: V. vinifera* glutathione S-transferase U10-like; VvGSTU10) was found co-expressed with STS (Martínez-Márquez et al., 2017). GSTs are required for the trafficking and vacuolar accumulation of anthocyanins (Marrs et al., 1995; Alfenito et al., 1998; Kitamura et al., 2004; Li et al., 2011; Sun et al., 2012; Conn et al., 2008; Gomez et al., 2011), most likely acting as a carrier or “ligandin” rather than a GSH-conjugating enzyme (Mueller and Walbot, 2001). Functional analysis of VvGSTU10 was shown to promote *t*-R transport to the extracellular medium in stably overexpressing grapevine cells (Martínez-Márquez et al., 2017), the first protein discovered involved in this role.

Different types of ATP binding cassette (ABC) proteins including multidrug resistance (MDR), multidrug resistance-associated protein (MRP), and pleiotropic drug resistance (PDR), as well as H^+^-gradient energized multidrug and toxic compound extrusion (MATE) transporters carry out secondary metabolite transport (reviewed in Grotewold, 2004; Yazaki, 2005; Lefèvre and Boutry, 2018; Dahuja et al., 2021). For instance, the alkaloids berberine, catharantine, and vincamine (Shitan et al., 2003a, 2003b; Yu and De Luca, 2013; Demessie et al., 2017); the terpenes sclareol, cembrene, and caryophyllene (Jasiński et al., 2001; Crouzet et al., 2013¸ Fu et al., 2017); and the phenolics anthocyanins, glucosylated anthocyanidin, p-coumaryl alcohol, liquiritigenein, and 4-coumarate (Gomez et al., 2011; Alejandro et al., 2012; Francisco et al., 2013; Banasiak et al., 2013; Biala et al., 2017; Behrens et al., 2019) are among the secondary metabolites whose transporter proteins have been characterized.

The transcriptomic analysis of MBCD- and MeJA-elicited grapevine cells displayed several genes encoding proteins for transport across membranes of ABC- and MATE-type upregulated and co-expressing with STSs (Almagro et al., 2014); however, to date, no stilbene membrane transporter has been characterized. Here, with the aim of investigating potential transporter candidates involved in the transport of *t*-R across membranes, we carried out a label-free proteomics analysis of plasma membrane and tonoplast fractions of 72-h-elicited grapevine cell cultures. One candidate, VvABCB15 (VIT_214s0066g02320|abc transporter b family member; LOC100854950–ABC transporter B family member 15; VIT_00032578001), was cloned and functionally analyzed. *In vitro*, *t*-R transport was assessed in transformed yeast microsomes. Moreover, the heterologous system of VvSTS-expressing *Silybum marianum* transgenic cell line (Hidalgo et al., 2017) transformed with VvABCB15 showed extracellular *t*-R levels higher than controls. The functionality of the VvABCB15 transporter was also analyzed in a homologous system by transient expression in *Vitis* cell cultures. Both elicited and non-elicited transiently transformed lines displayed a more significant extracellular *t*-R accumulation, thus providing the first conclusive evidence of the involvement of VvABCB15 in *t*-R transport to the extracellular medium. Confocal microscopy studies of *N. benthamiana* leaf showed that a transiently expressed VvABCB15-YPF fusion protein displays plasma membrane localization. The physiological and biotechnological relevance of the results is discussed.

## Materials and methods

### Plant material

Drs. J. C. Pech and A. Latché (ENSA, Toulouse, France) kindly supplied *V. vinifera* L. cv. Gamay calli in 1989. This cell line was maintained as solid and liquid cultures in Gamborg B5 medium as described elsewhere (Bru et al., 2006). *Nicotiana benthamiana* plants were obtained from seeds germinated and grown on potting soil in a greenhouse at 25°C, with a 16-h light/8-h dark photoperiod, until they were 3–5 weeks old.

VvSTS3-expressing (Ref. Seq. XM_002263686.2, PREDICTED: stilbene synthase 3 [*Vitis vinifera*]) *S. marianum* transgenic cell line (Hidalgo et al., 2017) was used for stable transformation experiments with VvABCB15. This cell line was maintained as solid and liquid cultures in MS medium as described elsewhere (Sánchez-Sampedro et al., 2005; Hidalgo et al., 2017).

### MRM experiments on protein subcellular markers

Samples for multiple-reaction monitoring (MRM) experiments were prepared as described in **Supplementary Methods**. The MRM acquisition method was based on surrogate tryptic peptides markers for *Arabidopsis* organelles (Parsons and Heazlewood, 2015; Hooper et al., 2017) suitably adapted to *V. vinifera* protein homologs using Skyline v19.1 software (MacLean et al., 2010). Target marker proteins, their surrogate peptides, and transitions for monitoring are schematized in **Supplementary Table S1**. MRM analysis was conducted in an Agilent 1290 Infinity LC coupled through an Agilent Jet Stream^®^ interface to a 6490 QQQ triple quadrupole mass spectrometer operated in the positive ion mode. Instrument settings include a spray voltage of 3.0 kV, a nebulizer (psi) of 30, an ion source temperature of 150°C, a gas flow of 15 L/min, and a cell acceleration voltage of 4 V. For each transition (**Supplementary Table S1**), the collision energy applied was optimized to detect the highest possible intensity. The peptide separation was achieved on an Agilent advance Bio Peptide Mapping column (2.1 × 150 mm, 2.7 µm) using a 7-min linear gradient of 3%–50% acetonitrile containing 0.1% (v/v) formic acid at a constant flow rate of 0.4 mL/min and an injection volume of 5 µL.

### Label-free proteomic analysis

A proteomic experiment was carried out using triplicates of the tonoplast and the plasma membrane-enriched fractions (see **Supplementary Methods**) from the grapevine cell suspension control and treated with 50 mM MBCD+0.1 mM MeJA elicitors for 72 h. Thirty micrograms of the desalted peptide digests were injected directly in Agilent 1290 Infinity UHPLC coupled through an Agilent Jet Stream^®^ interface to an Agilent 6550 iFunnel Q-TOF mass spectrometer (Agilent Technologies) system. Peptides were separated in a reverse-phase Agilent AdvanceBio Peptide mapping column (2.1 mm × 250 mm, 2.7 μm particle size, operating at 50°C) using a 140-min linear gradient of 3%–40% ACN in 0.1% formic acid at 0.400 mL/min flow rate. As detailed in **Supplementary Methods**, the mass spectrometer was operated in high-sensitivity mode.

Each MS/MS spectrum was preprocessed with the extraction tool of Spectrum Mill Proteomics Workbench (Agilent) to obtain a peak list and to improve the spectral quality by merging MS/MS spectra with the same precursor (Δ*m*/*z* < 1.4 Da and chromatographic Δ*t* < 15 s). The reduced dataset was searched against the proteome database of the PN40024 V2 (CRIBI) genome assembly (Vitulo et al., 2014) and contaminant proteins in the identity mode with the MS/MS search tool of Spectrum Mill Proteomics Workbench and with the following settings: trypsin, up to two missed cleavages, carbamidomethylation of Cys as fixed modifications, oxidation of Met, deamidation of Asn and Gln, and pyroGlu as variable modification and a mass tolerance of 20 ppm for precursor and 50 ppm for product ions. Peptide hits were filtered for a score ≥ 6, and percent scored peak intensity (%SPI) ≥ 60.

The analysis of protein differential abundance was determined using Progenesis QI for Proteomics (Nonlinear Dynamics) v4.0 label-free analysis software as detailed in **Supplementary Methods**.

### Functional analysis in yeast cultures

The VvABCB15 transporter (VIT_214s0066g02320; VIT_00032578001; LOC100854950) gene coding sequence codon-optimized for *N. benthamiana* was synthesized, supplied, and inserted into the pESC-URA-cMyc vector (GeneScript; Piscataway NJ, USA). *S. cerevisiae* strain CEN.PK 135 (ura-) was transformed with the pESC-URA-VvABCB15-cMyc vector by the LiAc/SS-DNA/PEG method (Gietz and Woods, 2002).

For *in vitro* transport studies with yeast microsomes, transformed yeast cells were grown overnight in YNB minimal medium without uracil, and spheroplasts were obtained by digestion with lyticase as described elsewhere (Tommasini et al., 1996). Briefly, yeast cells were cultivated overnight in YNB medium without uracil supplemented with 2% glucose at 30°C. The cells were sedimented at 1,200×*g* for 10 min, washed twice with water, and resuspended in 1.1 M sorbitol, 20 mM Tris-HCl (pH 7.6), and 1 mM DTT containing 57 units of lyticase per milliliter. After 90-min digestion at 30°C with gentle shaking, the spheroplasts were pelleted by centrifugation for 10 min at 1,200×*g* and lysed in a Dounce tissue homogenizer in 1.1 M glycerol, 50 mM Tris-ascorbate, pH 7.5, 5 mM EDTA, 1 mM DTT, 1.5% (w/v) PVP, 2 mg/mL bovine serum albumin, 1 mM PMSF, and Sigma Protease inhibitor cocktail. Unbroken cells and cell debris were removed by centrifugation at 4,000×*g* for 10 min at 4°C. The supernatants were centrifuged at 100,000×*g* for 45 min at 4°C. The membrane fraction was resuspended in 1.1 M glycerol, 50 mM Tris-Mes, pH 7.5, 1 mM EDTA, 1 mM DTT, 2 mg/mL bovine serum albumin, 1 mM PMSF, and Sigma Protease inhibitor cocktail. To study the transport of *t*-R into microsomes, uptake experiments were performed by using the rapid filtration technique (Tommasini et al., 1996) with nitrocellulose filters (0.45 µm pore size, Millipore) as described by Francisco et al. (2013). Briefly, 0.2 mL of yeast microsome suspension (200–350 µg total protein) was mixed with ice-cold transport solution (0.4 M glycerol, 100 mM KCl, and 20 mM Tris-HCl, pH 7.4) and freshly added 1 mM DTT, 5 mM MgSO_4_, 100 µg/mL creatine kinase, and 10 mM creatine phosphate. A *t*-R and MBCD stock solution in transport buffer was added to give a final concentration of 0.15 mM *t*-R and 2.5 mM MBCD, and transport was assayed in the absence and presence of 5 mM Mg ATP and 5 mM GSH in a total reaction volume of 0.8 mL. The transport mixture was incubated at room temperature for 30 min. Then, 0.4 mL of the mixture was immediately loaded on a prewetted filter and rapidly washed with 3 × 5 mL of ice-cold transport buffer supplemented with 20 mM MBCD. The filter-bound stilbenes were solubilized by incubating the filter in absolute methanol and extracting it for 30 min with shaking and analyzed as explained below.

For quantitative determination of the *t*-R efflux outside cells, the harvested yeast cells from an overnight culture grown to OD_600nm_ = 0.8 were incubated in the presence of 0.2 mM *t*-R for 4 h at room temperature with gentle shaking, followed by recovery by centrifugation for 10 min at 1,200×*g* at 4°C and washing the pellet with cold YNB medium. Time-course efflux experiments were performed by immediately transferring *t*-R-loaded yeast cells into fresh YNB minimal medium without uracil and incubating for 5, 10, and 15 min at room temperature, followed by centrifugation at 10,000 rpm for 10 min at 4°C in a benchtop microcentrifuge. Stilbenes in the supernatant were analyzed as explained below.

### Cloning of VvABCB15 transporter and VvSTS3 genes

Constructions for constitutive expression of eGFP, VvGSTU10, and VvSTS3 (**Supplementary Figures S2A–C**, respectively) are described elsewhere (Martínez-Márquez et al., 2015, 2017; Hidalgo et al., 2017). New DNA constructs for gene expression in plants were designed and assembled using the application GoldenBraid 3.0 standard for modular cloning (https://gbcloning.upv.es/) and cloning parts of the GoldenBraid 2.0 kit (https://www.addgene.org/Diego_Orzaez) (Sarrion-Perdigones et al., 2013). The experimental procedure is detailed in **Supplementary Methods**.

The binary vectors transformed chemically competent *Agrobacterium tumefaciens* strain EHA105 (Hood et al., 1993) by standard techniques (Sambrook et al., 1989).

### Subcellular localization of VvABCB15 in *Nicotiana benthamiana*

*A. tumefaciens* strain carrying the binary vector pDGB-VvABCB15-YFP (**Supplementary Figure S2F**) was used for transient expression by agroinfiltration (Schöb et al., 1997) co-infiltrated with a strain bearing cyan fluorescent protein (CFP) as fusion with plasma membrane aquaporin PIP2A (Nelson et al., 2007) for plasma membrane targeting and a strain containing the HC-Pro silencing suppressor (Goytia et al., 2006) in a 1:1:2 ratio in *N. benthamiana* leaves. Three to four days after agroinfiltration, the infiltrated leaves were harvested and analyzed by confocal microscopy (Leica TCS SP2; Leica Microsystems, Wetzlar, Germany). Leaf segments were cut, and abaxial leaf sides were scanned. An argon laser at 514 nm was used to excite the YFP. For visualization, the emission windows were set at 500–545 nm. Serial optical sections were obtained at 1-µm intervals, and projections of optical sections were accomplished with the Leica confocal software. Brightness and contrast were adjusted by Adobe Photoshop 7.0. ImageJ with the plugin JACoP was used to determine the Pearson correlation coefficient.

### Stable transformation of STS-expressing *Silybum marianum* transgenic cells

The *A. tumefaciens* containing pDGB-VvABCB15 (**Supplementary Figure S2D**) was used to stably transform the VvSTS3-expressing *S. marianum* transgenic cell line following the protocol described by Hidalgo et al. (2017). The transformed callus lines were established from individual calli growing in selection medium with 100 mg/L paromomycin. Sufficient callus material was obtained within 2–3 months of the initial transformation to check for plant genome T-DNA integration of the VvABCB15 gene by PCR amplification. The specific primers Fw 5′-ATGAACTCTTCCTTACAGGTGCC-3′ and Rev 5′-TGAGGTGCCTTGATGATTGC-3′ were used for amplifying a 1,097-bp fragment of the VvABCB15 coding region. The amplification reactions are as follows: 1 cycle at 95°C for 5 min and 30 cycles at 94°C for 20 s, 54°C for 30 s, and 72°C for 4 min, followed by an extension cycle of 10 min at 72°C. One transgenic callus was randomly selected, and, as shown in **Supplementary Figure S3**, the VvABCB15 gene was present in the transgenic line but not in the parental VvSTS3-expressing *S. marianum* line. One selected transgenic callus material was used to establish rapidly growing cell suspensions.

### Transient transformation of grapevine cells

*A. tumefaciens* harboring constructs (**Supplementary Figure S2**) were used to transiently transform *Viti’s* cell suspensions following the protocol described by Martínez-Márquez et al. (2017), but with a 3- or 6-day co-culture and no selection steps. The strains harboring the binary plant vectors pJCV52-VvSTS3 (Hidalgo et al., 2017) (**Supplementary Figure S2C**), pJCV52-VvGSTU10 (Martínez-Márquez et al., 2017) (**Supplementary Figure S2B**), and pDGB-VvSTS3 (**Supplementary Figure S2E**) were used alone or mixed with a strain containing pDGB-VvABCB15 (**Supplementary Figures S2A, D**) in a 1:1 ratio in *Vitis* cell cultures. From 3 or 6 days after *Agrobacterium*-infected, stilbenes content was analyzed.

### Elicitor or adsorbent compound treatments

In *Vitis*: Treatments were carried out in triplicate as previously described (Bru et al., 2006; Martinez-Esteso et al., 2011; Martínez-Márquez et al., 2017). Briefly, a weighted amount of filtered and washed cells was transferred into shaking flasks and suspended in fresh growth medium (4 mL/g of cell FW) supplemented with either elicitor (5 mM MBCD or 50 mM MBCD + 0.1 mM MeJA) or adsorbent compounds [1.5 g/L PVP or β-cyclodextrin (βCD)]. The cell suspension was incubated with continuous rotary shaking (100 rpm) at 25°C and under a 16-h light/8-h dark photoperiod.

In *Silybum*: Treatments were carried out in triplicate as previously described (Sánchez-Sampedro et al., 2005; Prieto and Corchete, 2014; Hidalgo et al., 2017). Briefly, 3 g of wet-weight 14-day cells was transferred to 100-mL flasks containing 20 mL of growth medium and incubated for 3 days before adding MBCD-containing medium to a 5 mM final concentration. Cultures were incubated in the dark at 25°C and shaken at 90 rpm.

### Determination of stilbenoids

Samples of extracellular and intracellular stilbenes of *Vitis* cell culture were prepared as described by Martínez-Márquez et al. (2016). Then, targeted quantitative analysis of stilbenoids by MRM was performed as described by Hurtado-Gaitán et al. (2017).

Extracellular *t*-R of *Silybum* cell culture was extracted three times with two volumes of ethyl acetate as described by Hidalgo et al. (2017). *t*-R analysis was performed by HPLC in a Spherisorb ODS-2 (5 μm) reversed-phase column (4.6×250 mm) at 35°C. The mobile phase was a mixture of 34 volumes of methanol and 66 volumes of acetic acid:water (5:55 v/v) at 1 mL/min (Hidalgo et al., 2017). Chromatograms were acquired at 306 nm. Identification of *t*-R was achieved by comparison with a commercial standard and confirmed by LC-MS analysis under the same conditions as reported by Hidalgo et al. (2017). Concentrations of *t*-R were estimated using the standard curve generated by the pure compound.

## Results

### Enrichment of grapevine cells’ subcellular extracts and checking by MRM analyses

**Figure 1:**
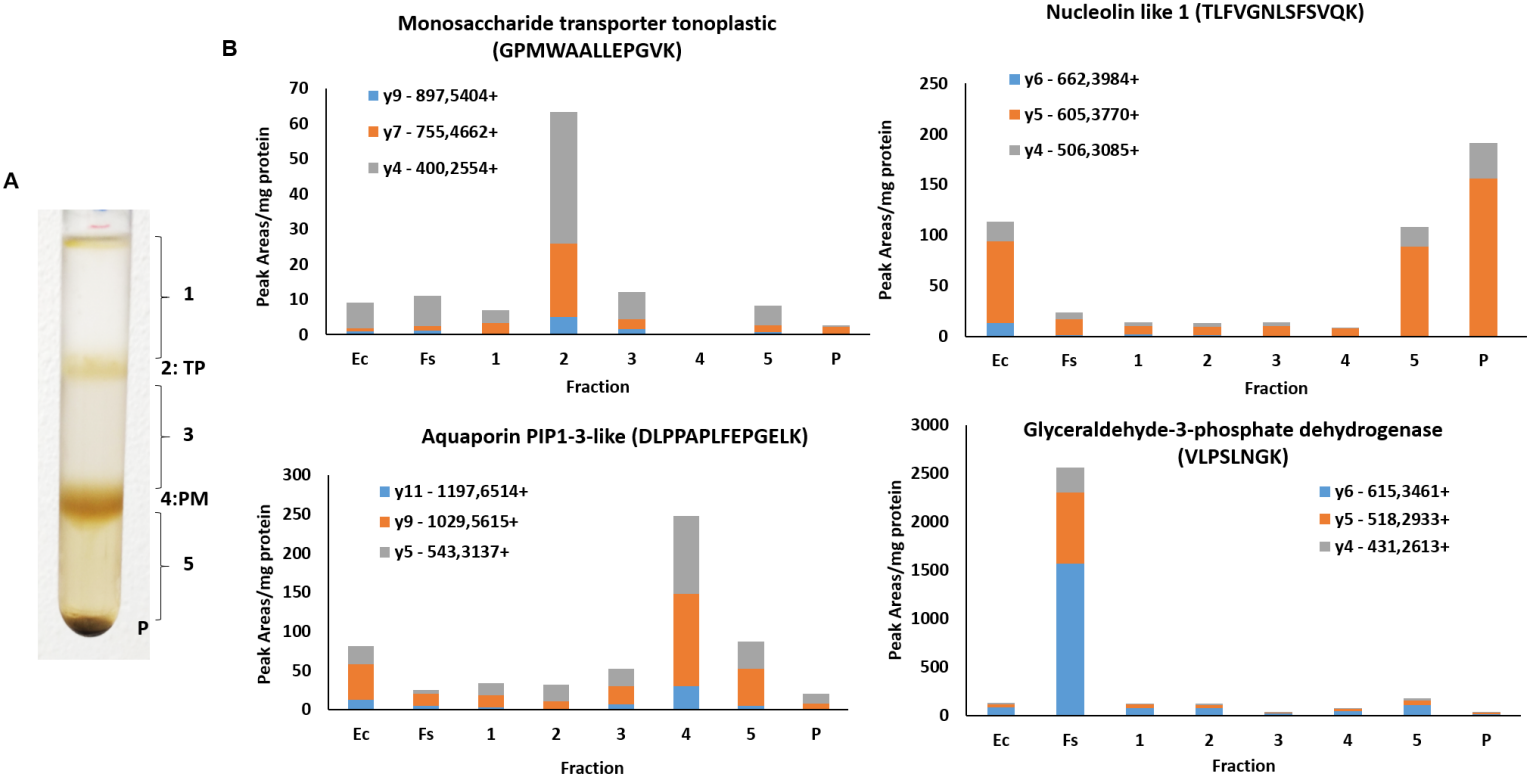
MRM assay in the subcellular fractions enriched by centrifugation density of grapevine microsomal fraction. **(A)** Gradient by centrifugation density of grapevine microsomal fraction. **(B)** Peaks areas of SRM transitions assay normalized with total protein concentration (mg protein) for the different fractions analyzed. Ec, crude extract; Fs, soluble fraction; TP, tonoplast; PM, plasma membrane.

One of the main objectives of this study is to discover candidate transporters involved in the mobilization of *t-*R and likely other monomeric stilbenes, which could be located in either the plasma membrane or the tonoplast. For this reason, obtaining fractions that are enriched in these organelles and as pure as possible is a critical step. For enrichment, checks of protein extracts and fractions, and Western or immunoblotting techniques are traditionally used using a set of specific antibodies against organelle-specific proteins. Unfortunately, antibodies for plant research have been developed for proteins of a few model plants; thus, their utility in most plant species relying on cross-reactivity is quite limited and scarcely validated. Alternative methods based on targeted proteomics, i.e., MRM, have been developed for Arabidopsis organelle marker proteins (Parsons and Heazlewood, 2015; Hooper et al., 2017) that surpass Western blotting in multiplexation of the analysis with similar sensitivity. Thus, we applied a specific MRM method based on the previously developed SRM (single-reaction monitoring) markers for Arabidopsis organelles suitably adapted to grapevine protein homologs to simultaneously detect and estimate relative organelle abundance, specifically vacuole, plasma membrane, cytosol, and nucleus. Target proteins were glyceraldehyde-3-phosphate dehydrogenase, monosaccharide transporter tonoplastic, nucleolin-like 1, and aquaporin PIP1-3-like, whose location is cytosol, tonoplast, nucleus, and plasma membrane, respectively. Specific tryptic peptides were initially selected for a unique protein of known localization (see supporting information in **Supplementary Table S1**). The method was tested on six subcellular fractions enriched by sucrose density gradient centrifugation of a grapevine microsomal fraction (**Figure 1A**) and two additional samples, crude extract (Ec) and soluble fraction (Fs).

**Figure 1B** shows peak areas of different SRM transitions of the study normalized by the total protein content in the fraction analyzed. According to the results, glyceraldehyde-3-phosphate dehydrogenase is more abundant in the soluble fraction as expected, and monosaccharide transporter tonoplastic is enriched in fraction 2 of the gradient, aquaporin PIP1-3-like in fraction 4, and nucleolin-like 1 in the crude extract and the heaviest fraction of the gradient and the pellet, as expected. These results confirm that the fraction enriched in tonoplast and plasma membrane are 2 and 4, respectively. Although there is some background of the plasma membrane in tonoplast, it represents barely 10% of the signal in fraction 4. Thus, according to the results of **Figure 1B**, it is more likely to detect some abundant plasma membrane protein in the tonoplast fraction than the opposite, i.e., tonoplast proteins in the plasma membrane fraction. Likewise, abundant nuclear or cytosolic proteins are found in the tonoplast or plasma membrane fractions but are poorly enriched compared to their correct organelle fraction. Thus, the MRM method successfully checked the enrichment in subcellular compartments of fractions prepared as described in **Supplementary Methods**.

### Identification of candidate *t*-R transporters

**Figure 2:**
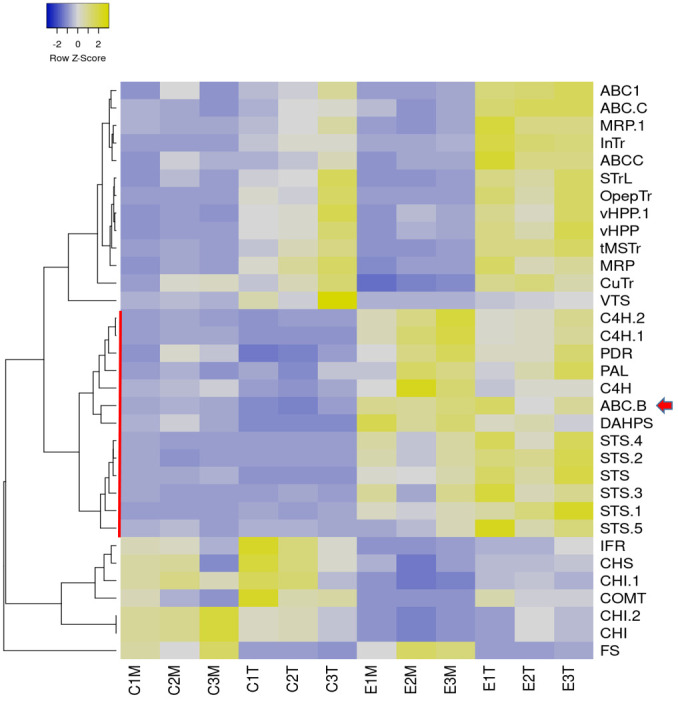
Label-free proteomic analysis of control (C#) elicited (E#) plasma membrane (M) and tonoplast (T) fractions. Normalized abundance heatmap of selected proteins filtered by ANOVA p<0,02 and fold change>4, involved in biosynthesis of stilbenes, flavonoids and lignans and transport across membranes: 3-deoxy-D-arabino- heptulosonate 7-phosphate synthase (DAHPS), phenylalanine ammonia-lyase (PAL), cinnamate-4-hydroxyIase (C4H), stilbene synthase (STS), chalcone synthase (CHS), chalcone isomerase (CHI), flavonol synthase (FS), isoflavone reductase homolog (IFR), caffeic acid O-methyltransferase (COMT), vacuolar H+-translocating inorganic pyrophosphatase (vHPP), tonoplastic monosaccharide transporter (tMSTr), ABC transporter B family member 15 (ABC B: VvABCB15), ABC transporter C family member 3-like and family member 4-like (ABC C), ABC1 family protein (ABC1), pleiotropic drug resistance protein 1-like (PDR), multidrug resistance protein MATE (MRP), sugar transporter erd6-like 6 (STrL), oligopeptide transporter 7-like (OpepTr), vesicle transport V-snare 13 (VTS), inositol transporter (InTr), copper transporter (CuTr). Figure prepared with Heatmapper (Babicki et al., 2016) using average linkage for clustering and Pearson as distance measurement method.

The grapevine cell cultures respond to MBCD and MeJA elicitor treatments with the continuous accumulation of extracellular *t*-R, reaching 3 g/L of culture and above, mainly as the trans-isomer, as described in previous studies (Lijavetzky et al., 2008; Martinez-Esteso et al., 2011; Martínez-Márquez et al., 2017). Quantitative proteomic analysis of whole-cell extracts has revealed the upregulation of t-R biosynthetic pathway enzymes PAL and STS as well as unanticipated elicitor-responding proteins such as tau class GST (Martinez-Esteso et al., 2011; Martínez-Márquez et al., 2017). The functional analysis of the latter provided evidence of the first protein involved in the transport of *t*-R to the extracellular medium (Martínez-Márquez et al., 2017). To broadly explore the expression profiles of proteins potentially involved in the *t*-R transport, a label-free proteomic experiment of enriched extracts in tonoplast or plasma membrane of 72 h elicited grapevine cell cultures was carried out (**Figure 2**) (**Supplementary Table S2**).

In total, 1,637 proteins were identified, of which 146 were found with significant differential abundance under the elicitation conditions (ANOVA *p* < 0.02 and fold change > 4) (**Supplementary Tables S3**, **S4**).

**Supplementary Figure S1** summarizes the label-free proteomic analysis of control and MBCD/MeJA-elicited plasma membrane (PM) and tonoplast (TP) fractions. Hierarchical clustering analysis of the abundance pattern distance (**Supplementary Figure S1A**) was set to classify the profiles into six color-coded groups (**Supplementary Figure S1B**). The same color code was used for the PCA bi-plot (**Supplementary Figure S1C**). The upregulated in elicitation treatment group vs. the control group (green) is the one that likely contains candidates for *t*-R transport across membranes. This green target group contained proteins involved in the biosynthesis of stilbenes (DAHPS, PAL, C4H, and STS), an ABC B class transporter, and a PDR protein belonging to the ABC G class transporter. Proteins that are competitors of STS for metabolic precursors (CHS, CHI, COMT, and IFR) belonged to the downregulated in elicitation treatment group (pink) or (FS) more abundant in the PM-enriched group (orange). To better select *t*-R transport candidates, we focused on proteins involved in the biosynthesis of stilbenes, flavonoids, and lignans and transport out of the 146 deregulated group. As seen in **Figure 2**, membrane transporters, including known tonoplast proteins (vHPP and tMSTr) and characterized (InTr, CuTr, OpepTr, and STrL) and uncharacterized transporters (ABC C, ABC1, and MRP-MATE), clustered as upregulated in tonoplast fraction with or without elicitation, while the ABC B and PDR transporters clustered with stilbene biosynthesis enzymes, upregulated in elicited groups.

**Figure 3:**
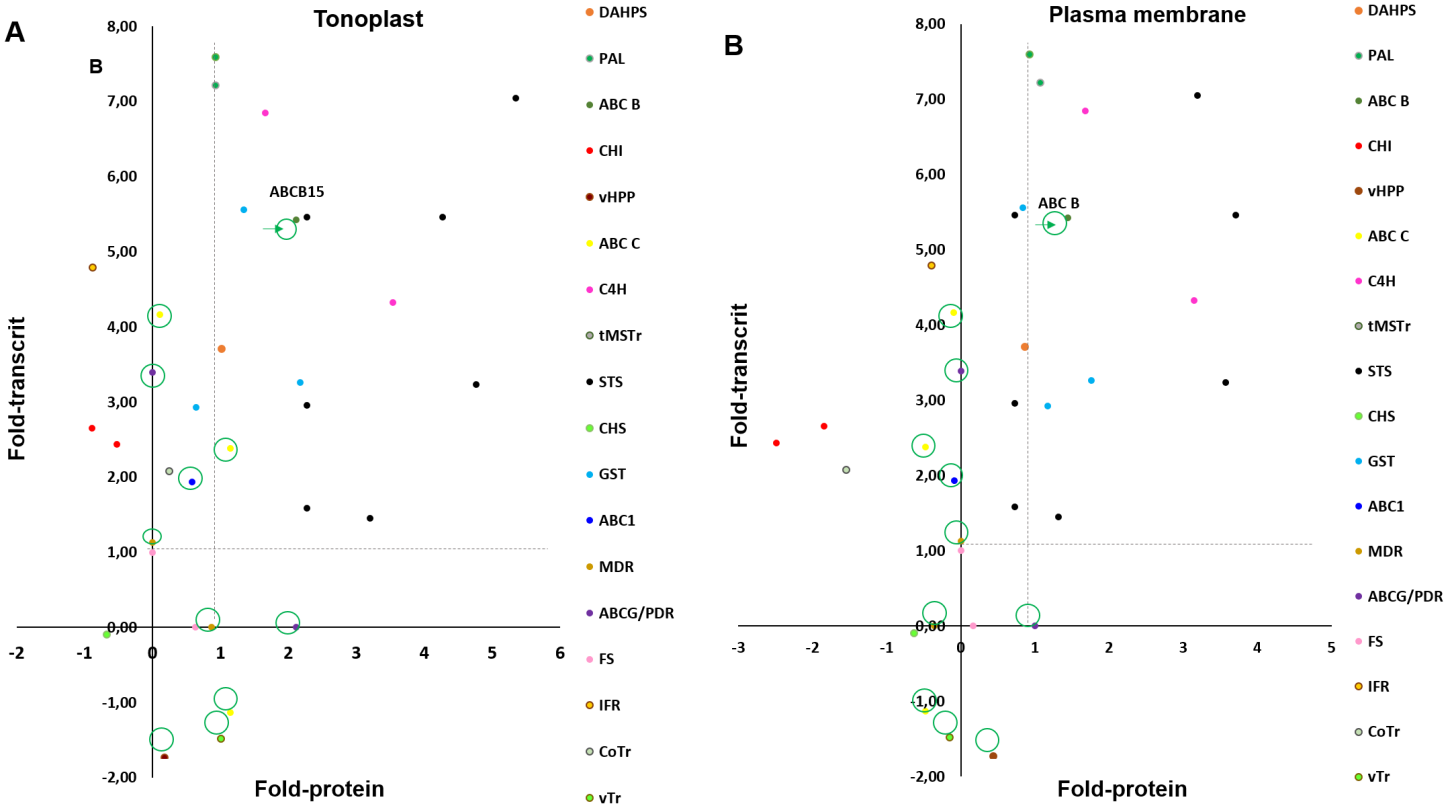
Comparison of transcript and protein changes in response to elicitation treatment with 50mM MBCD and 100µM. Transcript and protein fold-change presented as the Iog2 of the ratio of elicited: control conditions. Transcript fold change was obtained by microarrays in *Vitis Vinifera* cv. Monastrell at 24h of elicitation treatment (p<0,05 and fold change>2: see **Supplementary Information**, Almagro et al., 2015). Protein fold change was obtained by label-free proteomic analysis in tonoplast **(A)** plasma membrane **(B)** fractions of *Vitis Vinifera* cv. Gamay at 72h of elicitation treatment (p<0,02 and fold change>4; see **Supplementary Information**). Fold-change for biosynthesis of stilbenes, flavonoids and lignans and transport across membranes. 3-deoxy-D-arabino-heptuIosonate 7-phosphate synthase (DAHPS), phenylalanine ammonia-lyase (PAL), cinnamate-4-hydroxyIase (C4H), stilbene synthase (STS), gIutation-S-transferase (GST), chalcone synthase (CHS), chalcone isomerase (CHI), flavonol synthase (FS), isoflavone reductase homolog (IFR), vacuolar H+-translocating inorganic pyrophosphatase (vHPP), tonoplastic monosaccharide transporter (tMSTr), ABC transporter B family member 15 (ABC B: VvABCB15), ABC transporter C family member 3-like and family member 4-like (ABC C), ABC1 family protein (ABC1), pleiotropic drug resistance protein 1-like (PDR), multidrug resistance protein MATE (MRP), vesicIe transport V-snare 13 (vTr), copper transporter (CoTr). Fold changes correspondent to transport across membranes are marked with a circle.

According to these results, the ABC B and PDR are good candidates for the mobilization of *t*-R towards the extracellular medium in response to elicitors. To try to discriminate between both, we studied the correlation between the fold change at the level of protein (data obtained here) with that of transcripts, reported after 24 h of elicitation with MeJA + MBCD in *V. vinifera* cv Monastrell (Almagro et al., 2014). For comparison, we also included the GST U10-class involved in *t*-R extracellular accumulation (Martínez-Márquez et al., 2017). A separate comparison was made for each fraction, PM and TP, to cancel the effect of differential expression due to fractionation. As shown in **Figure 3**, transcript and protein for ABC B, as well as DAHPS, PAL, C4H, and STS, involved in stilbene biosynthesis, correlated positively; however, PDR transcripts encoding PDR protein were not found in the Almagro et al. (2014) report. Other transporters showed a poor correlation, likely due to the fraction cancellation effect.

Considering the above results, we selected the ABC B (VvABCB15) transporter for functional characterization.

### VvABCB15 localizes to the plasma membrane

To determine the subcellular localization of VvABCB15, the full-length gene was C-terminally fused to yellow fluorescent protein (YFP) and expressed under the control of the CAMV 35S promoter (P35S: VvABCB15-YFP). A plasma membrane aquaporin fusion PIP2A-CFP (for cyan fluorescent protein) was used as a control for plasma membrane localization (Nelson et al., 2007).

**Figure 4:**
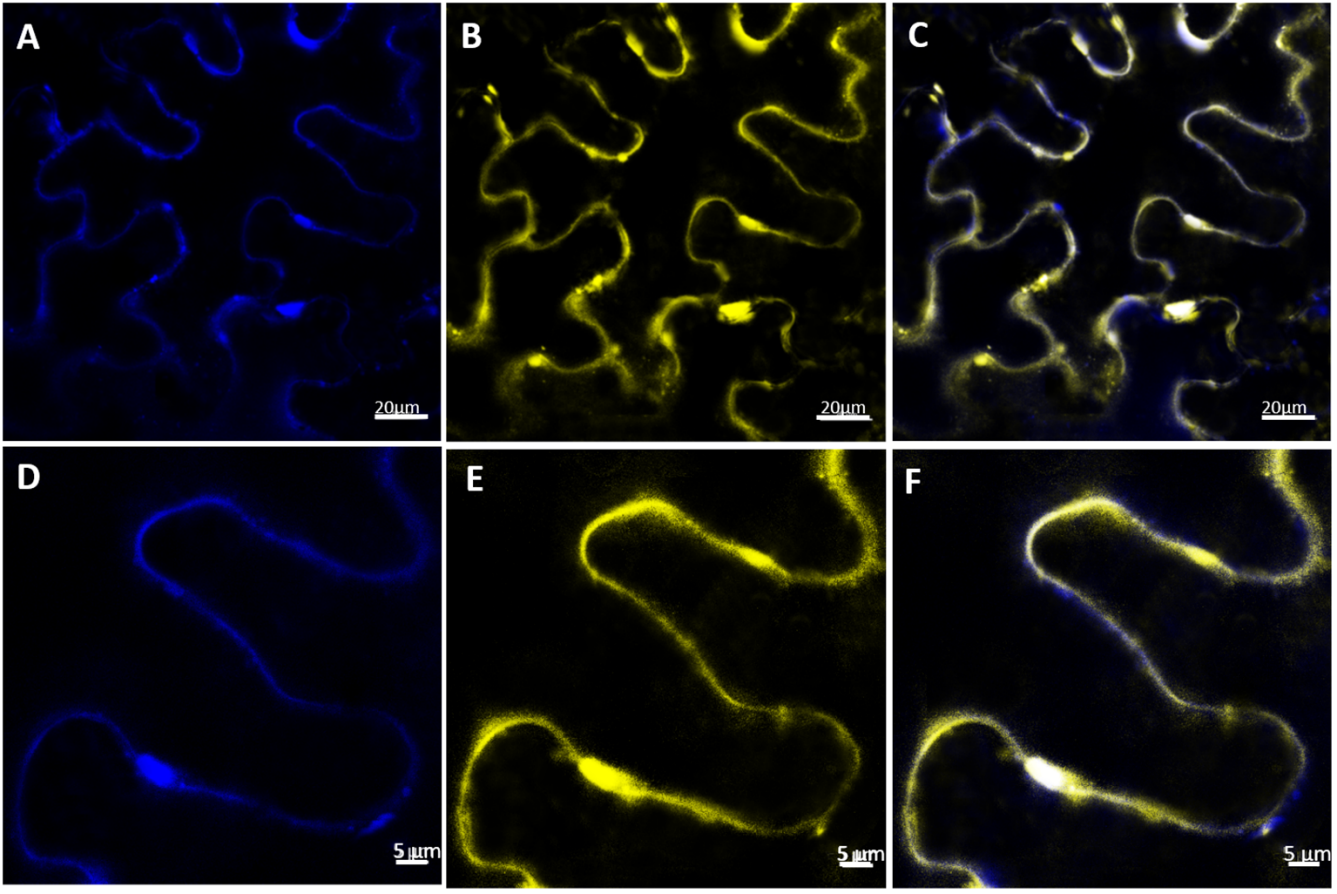
Subcellular localization of VvABCB15 transporter in *N. benthamiana* leaves by confocal microscopy four days after agroinfiltration. **(A, D)** PIP2-CFP expression in *N. benthamiana* leaves **(B, E)** VvABCB15-YFP expression in N. benthamiana leaves **(C, F)** merged images of one optical section.

Four-day agroinfiltrated *N. benthamiana* leaves observed under confocal microscopy showed the YFP signal as a band at the cell periphery, indicating that VvABCB15 could be located in the plasma membrane. As shown, the fluorescence distribution for both PIP2A-CFP (**Figures 4A, D**) and VvABCB15-YFP (**Figures 4B, E**) is found at the periphery of the cells when a single optical section is observed. Furthermore, merged images show that the two individual signals coincide almost exactly (**Figures 4C, F**), consistent with a plasma membrane colocalization (0.68 Pearson coefficient). Results provide clear evidence for plasma membrane localization of VvABCB15.

### Functional characterization of VvABCB15 as a *t*-R transporter in yeast cells

**Figure 5:**
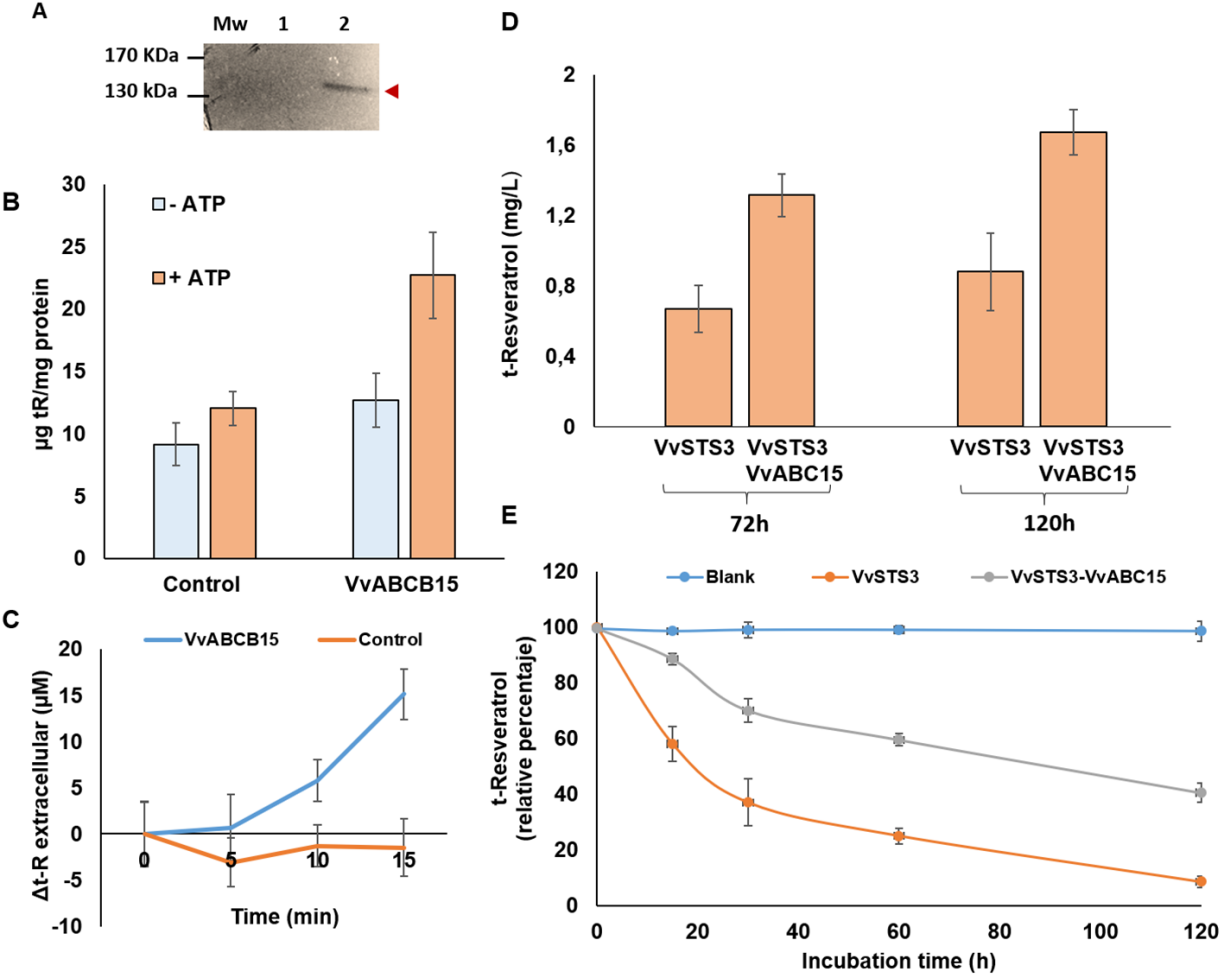
Functional analyses in yeast **(A–C)** and *Silybum marianum* **(D, E)** heterologous systems. **(A)** Expression of the VvABCB15 recombinant protein in yeast cells. Expression of the Myc-tag fusion proteins was confirmed by a Western blot analysis with an anti-Myc-tag antibody. **(B)** Net *t*-R uptake into microsomes isolated from yeast cells transformed either with the empty vector (control) or with VvABCB15 after 30 min incubated with 2.98mM of *t*- R in absence (blue) or presence (orange) ATP. Results are presented as mean values ± SE of two independent uptake experiments. **(C)** Extracellular efflux of *t*-R in yeast transformed cultures either with the empty vector (control) or with VvABCB15 after 4h incubated with 0.2mM of *t*-R. Data are the mean of three independent replicates ± SD. **(D)** Extracellular accumulation of *t*-R in the stable transgenic *S. marianum* cell suspensions in the presence of 5mM elicitor MBCD for 72h and 120 h. Data are the mean of three independent replicates ± SD. **(E)** Residual level of exogenously added *t*-R to STS- (orange) and STS+ABCB15- (grey) expressing *Sylibum marianum* transgenic cell lines. As control, residual level of *t*-R added to the medium but without cells (blue).

It is known that ABC transporters can transport a large number of chemically unrelated compounds (Kang et al., 2011). Following the observation that VvABCB15 co-expressed with *t*-R biosynthetic proteins in response to elicitors and that it is localized to the plasma membrane, we undertook the evaluation of its functionality as a stilbene transporter in both heterologous and homologous systems as an ultimate proof of its biological role. For this purpose, we ordered a synthetic version of the VvABCB15 gene cloned into the pESC-URA-cMyc plasmid for expression in *S. cerevisiae* fused in the C-terminal to a Myc tag. The presence of VvABCB15 in transformed yeast microsomal membrane vesicles was confirmed by Western blot with antiMyc antibody (**Figure 5A**). *t*-R is the most abundant stilbene in extracellular grapevine cell cultures, but also the cis isomer and dimers known as viniferins may accumulate to a significant extent (Lijavetzky et al., 2008; Martinez-Esteso et al., 2011; Martínez-Márquez et al., 2017). Thus, transport assays in yeast microsomes in the presence of *t*-R and MBCD as a *t*-R carrier were conducted using the rapid filtration technique reported by Tommasini et al. (1996). It is known that, in addition to the need for ATP for the functioning of ABC transporters, GSH is co-transported with the specific substrates in some cases (König et al., 2003). Thus, we performed the transport assays for studies with yeast microsomes, including both ATP and GSH, each at 5 mM final concentration, to preclude an eventual transport failure. The transport assays were also carried out without ATP as a functional control of VvABCB15. Targeted quantitative analysis of stilbenoids by MRM was used to quantify the stilbenes taken up into the vesicles.

In all the control assays, carried out with yeast microsomes that do not express the ABC transporter, we observed a transport “blank”, i.e., an amount of *t*-R retained in microsomes possibly due to either passive or nonspecific endogenous transport processes. That phenomenon has also been observable in the absence of ATP regardless of whether the assays are with yeast microsomes that express or do not express the ABC transporter (**Figure 5B**). In other studies, this phenomenon has also been reported, such as the transport of glycosylated anthocyanidins assayed in yeast microsomes (Francisco et al., 2013). However, significantly higher uptake of *t*-R into yeast microsomes expressing VvABCB15 was observed in the presence of ATP (**Figure 5B**). Also, controls without microsomes showed that a measurable amount of *t*-R bounded to the filter (2.8 ± 0.3 µg total) that was subtracted in both VvABCB15- and non-VvABCB15-expressing microsome assays. These observations in a heterologous system are the first proof of the functionality of the VvABCB15 transporter in the mobilization of *t*-R.

Since microsomes may proceed from membranes of different organelles and the orientation of the transporter in them may be random, the *in vitro* transport assay may provide inconclusive results about the transport directionality. Thus, we performed an *in vivo* assay of *t*-R efflux to the extracellular medium in yeasts after passive loading by 4-h incubation with 0.2 mM *t*-R. As seen in **Figure 5C**, VvABCB15-expressing yeast cells accumulate extracellular *t*-R in a time-dependent manner for 15 min. In contrast, in control cells, extracellular *t*-R does not change, remaining constant at a basal level of approximately 30 µM (**Supplementary Figure S4**). These results add strong evidence of the possible role of VvABCB15 in *t*-R efflux.

### Functional analyses by heterologous stable expression in VvSTS3-expressing *S. marianum* cell culture

The VvSTS3-expressing *S. marianum* transgenic cell line represents a heterologous system for *t*-R production under conditions of MBCD elicitation (Hidalgo et al., 2017). For this reason, and to evaluate the functionality of the VvABCB15 transporter in a plant heterologous system, this transgenic cell line was transformed with the construction harboring a TU for VvABCB15 (**Figure 4D**).

Control, STS3-expressing, and test, doubly expressing VvSTS3+VvABCB15, *S. marianum* cell suspensions were incubated in the absence and presence of 5 mM MBCD. As seen in the chromatograms of the extracellular media, *t*-R and an uncharacterized viniferin dimer can be detected in non-MBCD-treated suspensions as tiny peaks in control (**Supplementary Figure S5A**) but as prominent peaks in STS3+VvABCB15 (**Supplementary Figure S5B**). On the other hand, MBCD treatment strongly promotes the accumulation of these compounds in the extracellular medium, as Hidalgo et al. (2017) described. Still, the *t*-R and viniferin peaks are also more prominent in STS3+ABCB15 than in the control. **Figure 5D** shows the total accumulation of extracellular *t*-R after 72 and 120 h in the presence of 5 mM MBCD in the VvSTS3+VvABCB15 transformed line and VvSTS3 control line. As expected from the quantitative analysis of chromatograms, accumulation in the VvSTS3+VvABCB15 transformed cell suspensions was approximately 1.9-fold higher in each MBCD treatment than in control.

Likewise, it has been reported that *t*-R added to a grapevine cell suspension disappears after some hours (Morales et al., 1998) due to cell uptake and metabolism. Here, *t*-R was added externally to *S. marianum* cells expressing VvSTS3 or VvSTS3+VvABCB15 to follow its evolution with time under the hypothesis that an active outwards transport by VvABCB15 would secrete the *t-*R took up by cells, thus keeping its extracellular level higher than in control STS3-expressing cells. As seen in **Figure 5E**, that hypothesis was confirmed. Taken altogether, these results obtained in the *S. marianum* heterologous system provide further evidence of the functionality of VvABCB15 in the transport of free *t*-R.

### Functional analyses by homologous transient expression in *Vitis* cell culture

#### Functional analyses in the presence of t-R adsorbents

**Figure 6:**
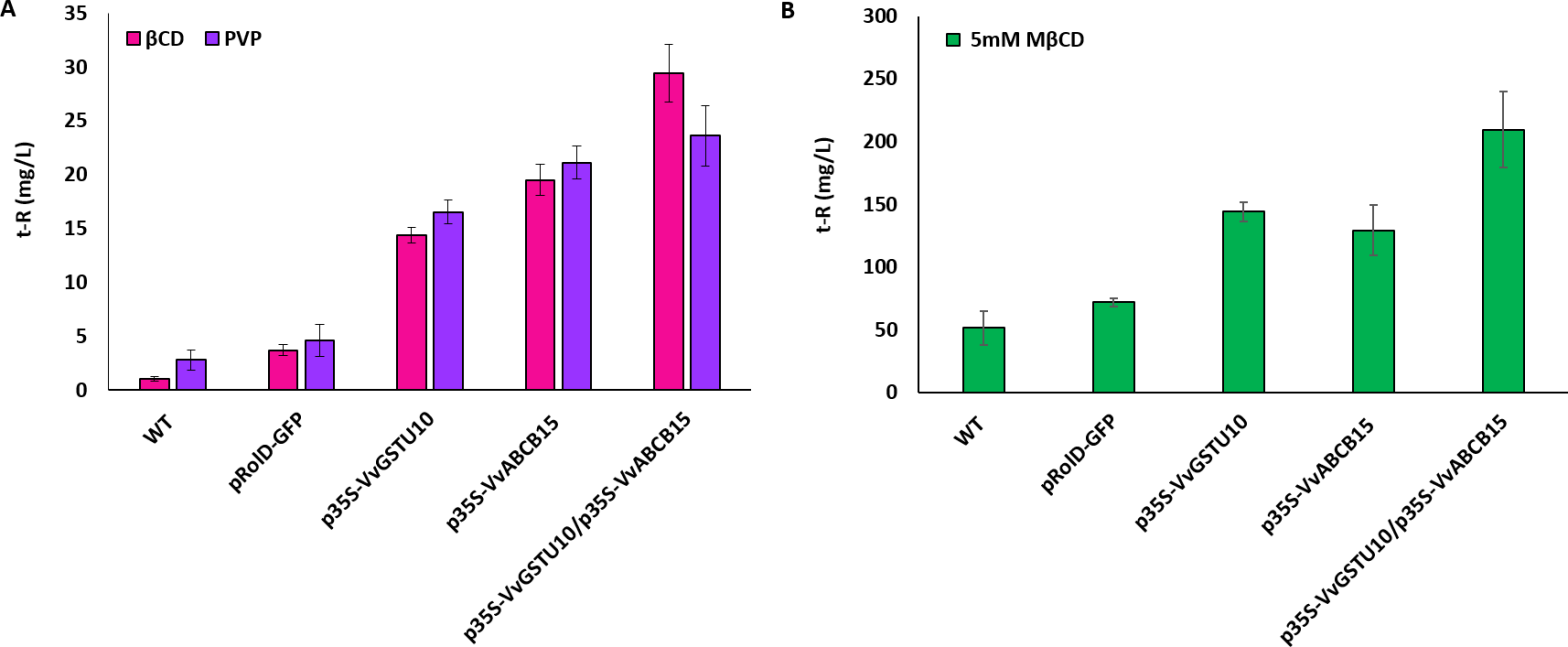
Extracellular accumulation of *t*-R in grapevine cell suspensions transiently transformed in the presence of PVP or BCD **(A)** and elicitor MBCD **(B)**. Effect of absorbent compound PVP (blue) and BCD (pink) on the extracellular *t*-R accumulation at 5 days after agroinfectation. Effect of 5mM elicitor MBCD (green) on the extracellular *t*-R accumulation for 72h after agroinfectation. Data are the mean of three independent replicates ± SD.

The increased extracellular accumulation of *t*-R under non-elicited conditions of *V. vinifera* cv. Gamay cells was used as a functional assay for putative candidate genes/proteins involved in *t*-R transport out of the cell (Martínez-Márquez et al., 2017). Gamay cells can constitutively synthesize stilbenes (*c*-Piceid and *t*-Piceid, and a modest *t*-R) and store them within cells during normal growth conditions (Martinez-Esteso et al., 2011; Martínez-Márquez et al., 2017). The occurrence of an active stilbene transport system would lead to the presence of the stilbene compounds outside of the cells at a higher level, as it was shown for the stable overexpression of VvGSTU10 (Martínez-Márquez et al., 2017). Here, transient expression experiments were carried out in Gamay grapevine cell cultures in the presence of adsorbent compounds PVP or βCD without the elicitor effect (Bru et al., 2006; Martínez-Márquez et al., 2017) to stabilize the cell-secreted *t*-R. Five days after agroinfection of the cell culture, extracellular *t*-R content was analyzed (**Figure 6A**). Small amounts of *t*-R, below 5 mg/L, were detected in the extracellular medium of the wild cells and the transformation control green fluorescent protein (GFP)-expressing cells due to both the basal production of stilbenes in Gamay cells and the stabilizing effect of both PVP or BCD (Martínez-Márquez et al., 2017). In the positive transport control, VvGSTU10-expressing cells, *t*-R extracellular levels reached above 15 mg/L. The transient expression of VvABCB15 resulted in *t*-R extracellular levels above 20 mg/L, and the joint transient expression of both VvGSTU10+VvABCB15 led to even higher levels, between 25 and 30 mg/L. This result demonstrates that under non-elicitation conditions, *t*-R transport towards the extracellular medium occurred to a much greater extent in VvABCB15 transiently transformed than in control or wild cells and that co-expression of both VvGSTU10 and VvABCB15 increases the transport capacity of the grapevine cells as compared to their counterparts. Results are virtually similar, irrespective of the adsorbent compound used. This result is consistent with its localization in the plasma membrane and the *t*-R mobilization activity demonstrated in yeast microsomes.

#### Functional analyses under mild elicitation

It is well known that the use of MBCD as an elicitor, either alone or combined with MeJA, usually at 50 mM and 100 µM, respectively, strongly increases the expression of genes of stilbene biosynthetic pathway and genes involved in *t*-R transport such as VvGSTU10 (Almagro et al., 2014; Martínez-Márquez et al., 2017), leading to a continuous increase in extracellular *t*-R and steady-state levels in the intracellular compartments. Under mild elicitation conditions (5 mM MBCD), the effect of stable overexpression of VvGSTU10 on *t*-R extracellular accumulation could be clearly seen above the background effect of the elicitor (Martínez-Márquez et al., 2017). Thus, we also analyzed the VvGSTU10 and VvABCB15 transient expression in Gamay cells under mild elicitation. **Figure 6B** shows the amount of extracellular *t*-R accumulated after 72 h of incubation with 5 mM of MBCD. As expected from the elicitor activity of MBCD, abundant extracellular *t*-R was found in all cell suspensions tested. Still, accumulation in the wild type and GFP-expressing transiently transgenic cell suspensions were much lower than in the VvGSTU10, VvABCB15, or VvGSTU10+VvABCB15 transiently transformed cell suspensions. Differences in the accumulated *t*-R between transformation control GFP-expressing and wild type were not significant, reaching between 50 and 70 mg/L. Separate expression of either VvGSTU10 or VvABCB15 led to higher levels, between 130 and 140 mg/L, but without significant differences.

Interestingly, their co-expression causes a further increase, reaching above 200 mg/L. This result is entirely consistent with that obtained above under non-elicitation conditions. It highlights the finding that VvGSTU10 and VvABCB15 cooperate in the transport and extracellular accumulation of *t*-R in grapevine cells.

#### Functional analyses using a light-switchable promoter

**Figure 7:**
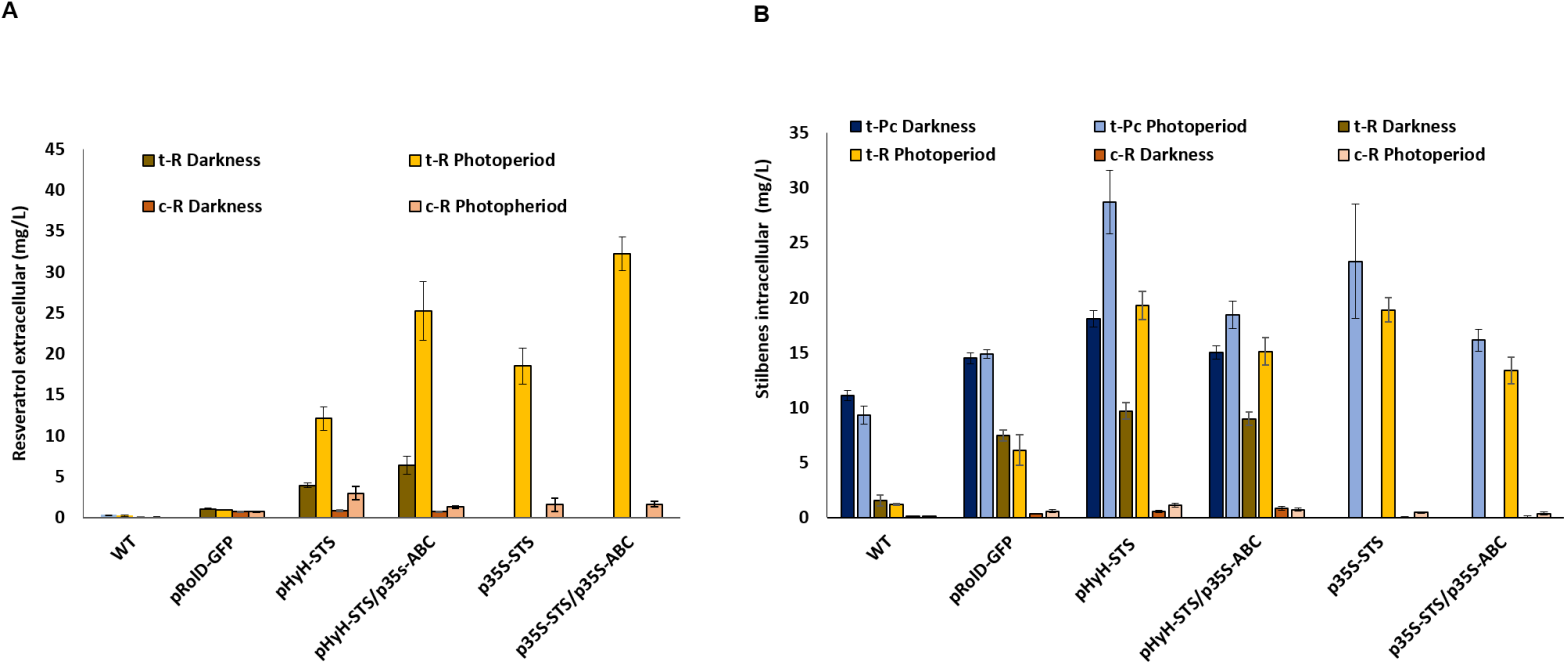
Stilbenes accumulation 5 days after agroinfection in grapevine cell suspensions transiently transformed. **(A)** Effect on the extracellular of trans-resveratrol (*t*-R) and cis-resveratrol (*c*-R) in darkness and photoperiod. **(B)** Effect on the intracellular of *t*-R, *c*-R and trans-piceid (*t*-Pc) in darkness and photoperiod. Data are the mean of three independent replicates ± SD.

Several studies have shown an increase in the accumulation of *t*-R upon the heterologous expression or overexpression of STS genes in different plant systems (Hain et al., 1990, 1993; Donnez et al., 2009; Kiselev and Dubrovina, 2021) occurring in the extracellular medium in case of cell or tissue cultures (Hidalgo et al., 2017; Chu et al., 2017). In this sense, quantitative changes in intra- and extracellular *t*-R in STS-overexpressing cells associated with the co-expression of a candidate can also be used as a functional assay for *t*-R transport. Here, we have overexpressed VvSTS1 and VvABCB15 under control of the constitutive promoter p35S in grapevine cells through transient transformation and quantified stilbenes inside and outside the cells by MRM in a multiplexed analysis, including *t*-R, *c*-R, and their glycosylated forms, i.e., piceid (*t*-Pc). In addition, VvSTS3 expression has also been handled using the light-switchable promoter pHyH (Zhang et al., 2023) to better dissociate the effects of infection from those of VvSTS1 overexpression on stilbene accumulation (see **Supplementary Figure S2** for constructs). Assays include two controls, namely, the wild-type culture and the transient expression of GFP under the pRolD promoter, the latter as a negative control of agroinfection. **Figure 7** shows the amount of the extracellular (**Figure 7A**) and intracellular (**Figure 7B**) stilbenoids accumulated after 5 days of infection either in darkness or under photoperiod. As mentioned above, wild-type Gamay cells produce constitutively mainly the glycosylated form piceid and a little of the free *t*-R, all intracellular. The transformation control expressing GFP shows a significant increase compared to wild-type, primarily free form in both cells and the extracellular medium. Wild-type or GFP-expressing cells do not show differences between darkness and photoperiod conditions. In the darkness, the stilbene accumulation in pHyH-VvSTS3 transformed cells is slightly higher than that in the transformation negative control. The above results indicate that the light regime has no background effect on stilbene production and that transformation itself causes a significant increase in the basal levels of free stilbenes and little impact on the glycosylated forms. In pHyH-VvSTS3 transformed cells, photoperiod conditions lead to a substantial rise in stilbene accumulation compared to darkness due to VvSTS3 expression. This result suggests that there must be constitutive transporters that facilitate the leaves of the readily VvSTS3-produced *t*-R. The co-transformation pHyH-VvSTS3/p35S-VvABCB15 compared to pHyH-VvSTS3 causes an increase in extracellular *t*-R in both conditions (1.64-fold for darkness and 2.1-fold for photoperiod) and, simultaneously, a decrease in intracellular stilbenes in both conditions, darkness (0.93-fold for *t*-R and 0.83-fold piceid) and photoperiod (0.77-fold for *t*-R and 0.64-fold piceid). The same phenomenon is observed when the expression of STS is constitutive, that is, co-transformation of p35S-VvSTS3/p35S-VvABCB15 compared to p35S-VvSTS3 causes an increase in extracellular *t*-R of 1.74-fold and, simultaneously, a decrease in intracellular stilbenes (0.71-fold for *t*-R and 0.69-fold piceid). These results can be interpreted as the effect of VvABCB15 overexpression that increases the rate of outward transport of the readily VvSTS3-produced free *t*-R, thus competing with the glycosylation reaction (supposedly unchanged) that keeps the *t*-R inside the cells as piceid. Consequently, the steady-state levels of *t*-R and the accumulated piceid decrease within cells while that of the extracellular free stilbenes increases.

*c*-R content did not change compared to the wild-type or GFP-expressing cells or changed only slightly for pHyH-VvSTS3 transformed cells intracellularly and for all VvSTS3 or VvSTS3+VvABCB15 transformed cells extracellularly, especially in photoperiod.

## Discussion

Phenolic secondary metabolites, such as stilbenes, play key biological roles in plant defense. They may preexist in high content or be synthesized after the microbial attack as part of constitutive and inducible defense responses, respectively (Chong et al., 2009). Their translocation to the extracellular compartment from the internal pools is essential as the first line of defense. This highlights the physiological importance of an efficient transport of stilbenes out of the cell.

Grapevine cell cultures show synthesis and accumulation of *t*-R in the extracellular compartment in response to an array of biotic and abiotic elicitors (Liswidowati et al., 1991; Calderon et al., 1993; Krisa et al., 1999; Belhadj et al., 2008; Yue et al., 2011; Tassoni et al., 2005; Ferri et al., 2009; Morales et al., 1998; Bru et al., 2006; Zamboni et al., 2006; Lijavetzky et al., 2008; Martinez-Esteso et al., 2009, 2011; Belchí-Navarro et al., 2012, 2013; Almagro et al., 2014, 2015), being ideal biological platforms for the study of resveratrol transport. In this sense, Martínez-Márquez et al. (2017) demonstrated the involvement of VvGSTU10 in the *t*-R transport to the extracellular medium in the grapevine cell culture elicited with MBCD combined with MeJA. However, despite their utmost physiological and biotechnological relevance, the transport pathways of *t*-R and other stilbenes to the extracellular medium in grapevine cells are entirely unknown. The present work is thus the first study to aim to find membrane transporters involved in this transport process.

The proteomic experiment on plasma membrane- and tonoplast-enriched fractions of 72-h-elicited grapevine cell cultures has allowed the discovery of several transporters that fit the expression profile of resveratrol biosynthetic enzymes (**Figure 2**). However, the comparison of transcript (Almagro et al., 2015) and protein changes in response to elicitation treatment with 50 mM MBCD and 100 µM MeJA (**Figure 3**) led us to select an ABC-B-type transporter, VvABCB15, as a target candidate for functional analysis.

Specifically, ATP-binding cassette (ABC) proteins are multidomain transmembrane proteins that use the energy obtained from ATP hydrolysis to translocate molecules like xenobiotics, hormones, sugars, amino acids, ions, primary and secondary metabolites, among others, involved in essential physiological processes such as nutrition, development, responses to biotic and abiotic stress, interaction with the environment, and mainly in transmembrane transport (Lefèvre and Boutry, 2018; Nogia and Pati, 2021). Generally, ABC transporters possess two types of domains, namely, cytosolic nucleotide-binding domain (NBD)/ATP binding domain and transmembrane domain (TMD) (Sharom, 2011). Based on the combination and number of TMDs/NBDs, the ABC transporter family has often been grouped into nine subfamilies, viz., ABCA, ABCB, ABCC, ABCD, ABCE, ABCF, ABCG, ABCH, and ABCI (Verrier et al., 2008), among which ABCH has not been identified in plants. Their involvement in the trafficking of secondary metabolites has been well demonstrated for alkaloids (Shitan et al., 2003a; 2003b), terpenes (Jasiński et al., 2001; Yu and De Luca, 2013; Demessie et al., 2017; Fu et al., 2017), phenolic compounds as glucosylated anthocyanidin (Francisco et al., 2013; Behrens et al., 2019) or monolignol (Alejandro et al., 2012), and volatile compounds (Adebesin et al., 2017). However, no membrane transporter has been described as a protein involved in the mobilization of stilbenes, specifically *t*-R.

So far, 120 putative ABC members have been identified in the grapevine (*V. vinifera*) (Çakır and Kılıçkaya, 2013). The main feature of the Vitis ABC superfamily is the presence of several large subfamilies, which include ABCG (PDR and white-brown complex homolog, PDR), ABCC (multidrug resistance-associated protein, MRP), and ABCB (multi-drug resistance/P-glycoprotein, MDR/PGP). The plant MRP/ABCCs subfamily has been proposed to be involved in the vacuolar sequestration of potentially toxic metabolites (Klein et al., 2006). Consistently, the expression profiles of ABCC subfamily transporters detected in our label-free proteomic experiment were higher in the tonoplast; thus, they were rejected as candidates for transporting stilbenes outside the cell. Members of the ABCG family are expressed in plants in response to various biotic and abiotic stresses and play important roles in detoxification processes, preventing water loss and transport of phytohormones and secondary metabolites (Alav et al., 2021). A PDR belonging to the ABC G class transporter, which fits the expression profiles of *t*-R biosynthetic enzymes, was discovered along with an ABC B class transporter. Because the transcript encoding that PDR protein was not found in Almagro et al.’s, 2014 report, we selected ABC-B transport as the only candidate involved in *t*-R transport outside *Vitis* cells. Only a few ABCB transporters have been extensively characterized in plants and shown to catalyze the transport of structurally diverse substrates, such as phytohormones, xenobiotics, and secondary metabolites (Bailly et al., 2012). Recently, genome-wide analysis has identified 19 ABCB members in the grapevine whose expression responds to berry development and iron and heavy metal stress (Çakır et al., 2023). Kaneda et al. (2011) showed that three ABCB and one ABCG gene whose expression correlated with phenylpropanoid biosynthetic genes and lignification in Arabidopsis were directly or indirectly related to secondary cell wall deposition or with lignification. Here, such ABCB and ABCG correlation with phenylpropanoid biosynthesis has been observed as well at the protein level in response to elicitation but, in this case, related to stilbene biosynthesis that, as lignin precursors, derives from phenylpropanoids. All these findings point to a molecular role of transport towards the extracellular medium or apoplastic space of certain ABCB members. The plasma membrane subcellular localization of VvABCB15 is highly consistent with that role, being supported by both the colocalization with the plasma membrane protein marker aquaporin PIP2A (Nelson et al., 2007) and the efflux of resveratrol to the extracellular medium in heterologous systems transformed with this grapevine transporter gene, such as yeast and *S. marianum* cells.

The functional characterization VvABCB15 was successfully performed in both heterologous and homologous systems. Wild yeast and *S. marianum* cell cultures stably expressing VvSTS3 were used as heterologous systems. Our results showed that resveratrol transport occurs more intensively in both VvABCB15-transformed heterologous systems than in their controls. In the case of yeast, this greater transport of resveratrol is only observed in the presence of ATP. Further strong evidence of the role of the VvABCB15 transporter in the mobilization of *t*-R to the external medium was obtained by *in vivo* studies of the evolution over time of *t*-R efflux from pre-loaded yeast cells.

In the case of the *S. marianum* heterologous system, previous studies showed that *S. marianum* cells expressing grapevine VvSTS3 and incubated with MBCD accumulated extracellular *t*-R (Hidalgo et al., 2017), which are the exact conditions we have used as control. It suggests that these cells have endogenous non-specific mechanisms to move *t*-R outwards, and thus the observed enhancement in the double transformants (VvSTS3+VvABCB15) can be attributed to the activity of the specific transporter. Moreover, the slower disappearance in the extracellular medium of *t*-R externally added to an *S. marianum* cell suspension expressing the transporter provides further evidence that the stable expression of VvABCB15 in this heterologous system gives rise to an active outward *t*-R transport whether the *t*-R is synthesized in the cell or whether it is taken up from the extracellular medium.

Here, we carried out two experiments using *V. vinifera* cv Gamay cell culture as a homologous system for *t*-R transport functional assays. The rationale behind it relies in its constitutive *t*-R synthesis capacity, which is almost totally accumulated inside the cells in its glycosylated form piceid. In addition, the *t*-R synthesis capacity was handled through the expression of VvSTS3 under control of the light-sensitive promoter pHYH (Zhang et al., 2023), which allowed us to regulate the amount of *t*-R available for transport and glycosylation. Thus, the transport of *t*-R outside the cells promoted by the expression of a candidate gene becomes a competing pathway with the glycosylation and storage that can be conveniently monitored by determining the level of extracellular *t*-R. The stable expression of VvGSTU10 in Gamay grapevine cells gave rise to the accumulation of extracellular *t*-R, thus demonstrating its involvement in *t*-R transport out of the cell (Martínez-Márquez et al., 2017). Here, we have obtained similar results but carried out transient expression instead, thus validating that this type of assay is much less time-consuming than stable expression. The effect of transient expression of VvABCB15 in the Gamay cell culture is similar to or slightly higher than that of VvGSTU10, and much higher than a mock gene such as GFP in all conditions tested, thus providing strong evidence for the involvement of this particular transporter in *t*-R transport out of cells. On the other hand, the co-expression of both VvGSTU10 and VvABCB15 further increased the transport capacity of the grapevine cells as compared to their individual counterparts pointing towards a cooperative action in *t*-R transport out of grapevine cells. Results obtained in cells expressing both VvABCB15 constitutively and VvSTS1 under control of pHYH and in different light conditions are highly consistent with the above, providing strong evidence of the role of VvABCB15 as a *t*-R transporter. When comparing the extra- and intracellular profile of stilbenes (**Figure 7**), it can be noticed that the increase of free stilbene *t*-R in the extracellular medium due to expression of the transporter (no matter whether it is light-inducible or constitutive) corresponds to a concomitant decrease inside of both the free and the glycosylated form piceid. This result can be explained by the competition between the transporter and the glycosylating enzymes for the free *t*-R within the cell and strongly supports the plasma membrane localization of VvABCB15 since if it would also localize in tonoplast, the internal *t*-R concentration should have increased as well.

The GST enzymes are long known to be involved in vacuolar accumulation of anthocyanins as well as ABCC-type transporters (Goodman et al., 2004), but as the formation of anthocyanin–GSH conjugates is not required for anthocyanin/GSH co-transporters such as VvABCC1 in the grapevine (Francisco et al., 2013) or AtABCC2 in Arabidopsis (Behrens et al., 2019), an accepted role of GST is acting as carriers or ligandins to deliver these compounds to the transporters (Sun et al., 2012). Although, currently, there are no data to support the effective cooperation between these proteins, one could speculate on different scenarios. On the one hand, these two proteins might act independently and carry out the transport by a parallel mechanism, producing additive effects. On the other hand, acting as ligandin as was proposed for VvGSTU10 (Martínez-Márquez et al., 2017), it would facilitate the movement of the poorly water-soluble *t*-R within the cell and bring it closer to the vicinity of the membrane for transport by VvABCB15, thus enhancing the transport rate by an increase of the local *t*-R concentration. Future experiments that determine proximity or even interaction between these proteins are needed to cast light on this issue.

From the work presented herein, it can be concluded that VvABCB15 is a plasma membrane transporter of *t*-R involved in the machinery that is not yet fully characterized for accumulating *t*-R in the extracellular medium as part of a defense response. To our knowledge, this is the first ABC transporter and the second protein, together with VvGSTU10 (Martínez-Márquez et al., 2017), described for this function, and future studies will help to elucidate whether other membrane transporters and pumps could be involved in the said machinery. Some other candidates have already been recognized in the proteomics experiment present in this study and by Almagro et al. (2014) and will be investigated in future work.

## Data Availability

The datasets presented in this study can be found in online repositories. The proteomics data presented in the study are deposited in the Pride repository, accession number: PXD048454. Label-free proteomics nálisis of plasma membrane and tonoplast fractions of elicited grapevine cell cultures.

## In memoriam

Dedicated in memoriam to our co-author, colleague and friend Purificación Corchete who passed away on December 19th, 2023.
