# Supplementary material for: The grapevine ABC transporter B family member 15 is a trans-resveratrol transporter out of grapevine cells
Source: Front Plant Sci. 2025 Jan 20;15:1450638. doi: 10.3389/fpls.2024.1450638 (PMC11792551; doi:10.3389/fpls.2024.1450638)
Supplement: Supplementary file 1 [file DataSheet1.zip › Supplementary Methods.docx]

**Supplementary Methods**

**Martínez-Márquez et al.,**

**Preparation of grapevine cells subcellular fractions and protein extracts**

Grapevine cells control and elicited with 50 mM MBCD and 0.1 mM MeJA were harvested after 72h incubation from liquid cell cultures by filtration under gentle vaccum. Subcellular fractions were obtained after mechanical lysis in a potter Elvehjem homogenizer of cell suspensions in 50mM HEPES pH 7.5, 0.25M sucrose,1% (w/v) PVPP, 5% (w/v) glycerol, 10mM EDTA, 10mM Na_2_O_5_S_2_, 10mM ascorbic acid, 1mM PMSF and Sigma Protease inhibitor cocktail at a ratio of 2mL per gram of plant material at 4 °C. Cellular debris was removed by centrifugation at 8000xg for 10min at 4°C and the supernatant (crude extract, Ec) ultracentrifuged at 100,000xg for 1:30h at 4°C. The supernatant (soluble fraction, Fs) was kept apart and the pellet was washed twice by resuspension in double volume of 50mM HEPES pH 7.5, 0.25M sucrose, 5% (w/v) glycerol, 10mM EDTA, 10mM Na_2_O_5_S_2_ and recovered by ultracentrifugation as above. The washed pellet was resuspended in 50mM HEPES pH 7.5, 5% glycerol and layered over a discontinuous sucrose gradient (0, 23 and 32% w/v) prepared in polyallomer tubes and separated by ultracentrifugation at 100,000xg for 3h at 4ºC in a swing-out rotor.

Aliquots of the crude extract, the soluble fraction, and each different subcellular sucrose gradient fractions were precipitated as described by Granier and Van of Walle (1988), with slight modifications. Sample was brought to a volume of 750 µl by adding distilled water followed by 8.5 µl of 2% (w/v) sodium deoxycholate solution (Bensadoun and Weinstein, 1975); after mixing and 10 minutes of incubation on ice, 250 µl of 24% (w/v) TCA was is added, vortexed and incubated for 30 minutes on ice to quantitatively precipitate proteins. The protein pellet obtained by centrifugation at 14000xg for 10 min at 4°C was washed twice with chilled 10% (w/v) TCA in acetone followed by twice in pure chilled acetone. Finally, the clean protein precipitate obtained was let to dry at room temperature, solubilized in 6M urea and quantified by RC DC protein assay (BIO-RAD) (Raghupathi and Diwan, 1994). One hundred micrograms of precipitated protein sample were digested with trypsin and 30 µg of the resulting peptides desalted with PepClean C-18 Spin Columns (Agilent Technologies) according to manufacturer recommendations as previously described (Martinez-Marquez et al. 2013).

**Label-free proteomic analysis**

A proteomic experiment was carried out using triplicates of the tonoplast and the plasma membrane enriched fractions from the grapevine cell suspensions control and treated with 50 mM MBCD+0.1 mM MeJA elicitors for 72 hours. Thirty micrograms of the desalted peptide digests were injected directly in Agilent 1290 Infinity UHPLC coupled through an Agilent Jet Stream® interface to an Agilent 6550 iFunnel Q-TOF mass spectrometer (Agilent Technologies) system. Peptides were separated in a reverse phase Agilent AdvanceBio Peptide mapping column (2.1 mm × 250 mm, 2.7 μm particle size, operated at 50 °C) using a 140 min linear gradient of 3-40 % ACN in 0.1 % formic acid at 0.400 mL/min flow rate. The mass spectrometer was operated in high sensitivity mode. Source parameters employed gas temp (250 °C), drying gas (14 L/min), nebulizer (35 psi), sheath gas temp (250°C), sheath gas flow (11 L/min), capillary voltage (3,500 V), fragmentor (360 V). The data were acquired in positive-ion mode with Agilent MassHunter Workstation Software, LC/MS Data Acquisition B.08.00 (Build 8.00.8058.0) operating in Auto MS/MS mode whereby the 20 most intense ions (charge states, 2 -5) within 300 - 1,700 m/z mass range above a threshold of 1,000 counts were selected for MS/MS analysis. MS/MS spectra (50 - 1,700 m/z) were collected with the quadrupole set to “narrow” resolution and were acquired until 25,000 total counts were collected or for a maximum accumulation time of 333 ms.

Each MS/MS spectra was preprocessed with the extraction tool of Spectrum Mill Proteomics Workbench (Agilent) to obtain a peak list and to improve the spectral quality by merging MS/MS spectra with the same precursor (Δm/z<1.4 Da and chromatographic Δt < 15s). The reduced dataset was searched against the proteome database of PN40024 V2 (CRIBI) genome assembly (Vitulo et al. 2014) and contaminant proteins in the identity mode with the MS/MS search tool of Spectrum Mill Proteomics Workbench and with the following settings: trypsin, up to 2 missed cleavages, carbamidomethylation of Cys as fixed modifications, oxidation of Met, deamidation of Asn and Gln, and pyroGlu as variable modification and mass tolerance of 20ppm for precursor and 50ppm for product ions. Peptide hits were filtered for score ≥ 6 and percent scored peak intensity (%SPI)≥60.

The LC-MS raw files were imported into Progenesis QI for Proteomics (Nonlinear Dynamics) v4.0 label-free analysis software. Quantification was done on the basis of MS1 intensity. The data file that yielded most features (peaks) was used as reference to align the retention time of all other chromatographic runs and to normalize MS feature signal intensity (area under the peak). Correction for experimental variations was done by calculating the robust distribution of all ratios (log(ratio)). The MS features were filtered to include only features with charge state from two to five. After defining the experimental design as “between subjects” mode samples were clustered according to the experimental groups (Control PM, Control T, elicited PM and elicited T) and the average intensity ratios of the matched features across the experimental groups as well as the p-value of one-way ANOVA were automatically calculated. To identify the proteins from which detected features come from, the filtered SpectrumMill peptide hits files were imported into Progenesis QIp, the peptide assignment conflicts resolved (the highest score the winner, or were left unresolved in case of equal score and sequence), and the inferred protein list filtered by score≥15. The abundance of proteins was automatically calculated by the Hi-3 method as described by Silva et al. (2006) implemented in the Progenesis QI for proteomics. Differential protein abundance across experimental group was assessed by using the advanced statistical tools implemented in Progenesis QIp including ANOVA, PCA and Power analysis.

**Western blotting**

The microsomal pellet was solubilised in 1x SDS-PAGE sample buffer and denatured at 90ºC for 5 min. The protein concentration was determined by an RC DC protein assay (BIORAD) (Raghupathi and Diwan, 1994). Proteins (30 µg/lane) were resolved by SDS-PAGE and electro-transferred to the Hybond-P PVDF membranes (GE Healthcare). Membranes were probed at 4◦C overnight with mouse monoclonal anti-Myc-Tag antibodies (Sigma) at the 1:1000 dilution, and were incubated at room temperature for 1 h with horseradish peroxidase-conjugated goat anti-mouse IgG at the 1:10000 dilution. Detection was performed by ECL using the Prime Western Blotting Detection Reagent SuperSignal West Dura system (GE Healthcare, Amersham).

**Cloning of VvABCB15 transporter and VvSTS3 genes by GoldenBraid 2.0 kit**

For domestication in the pUPD2 vector, the sequences of interest were PCR amplified flanked by its part category suffix and prefix, and BsmBI recognition sites, as well as removal of internal BsmBI and BsaI recognition sites (See supporting information, Table S5).

Transcriptional units (TU) consisting of a promoter, a coding sequence and a terminator were assembled into pDBG destination plasmids. VvABCB15 TU (pDGB-VvABCB15) consisted of the cauliflower mosaic virus (CaMV) 35S promoter (P35S, GB0030), the VvABCB15 synthetic sequence without the Myc tag and the nopaline synthase terminator (TNos, GB0037) (Figure 2-Suppl.D). In addition, to afford subcellular localization a VvABCB15 C-term fused to YFP TU (pDGB-VvABCB15-YFP) (Fig. 2-Suppl.F) under control of P35S and TNos was assembled. VvSTS3 TU (pDGB-VvSTS3) was built under control of the light responding HY5 HOMOLOG promoter pHYH (Zhang et al., 2023) from grapevine (kindly provided by Tomás Matus) and TNos terminator. A TU for aminoglycoside antibiotic selectable marker was built containing the neomycin phosphotransferase (NPTII, GB0226) coding sequence under control of Pnos and Tnos (Fig. 2-Suppl.D,E).

The GB2.0 α level-assembled transcriptional units (TUs) were further combined within Ω level destination vector backbones, yielding appropriate multigenic constructs as shown in Figure S2.

**SUPPORTING INFORMATION**

**Table S5:** Oligonucleotides used for GB2.0 domestication and amplification reactions.

**Figure S2:** Schematic diagram of the expression constructs used for transient transformation experiments. P35S, cauliflower mosaic virus (CaMV) 35S promoter; Pnos, nopaline synthase promoter; pHyH, HyH promoter; nptII, neomycin phosphotransferase; VvSTS3, Stilbene synthase; VvGSTU10, Glutathione-S-transferase; VVABCB15, ATP binding cassette B 15; eGFPer, Green fluorescent protein with ER signal peptide sequence; YFP, Yellow fluorescent protein, T35S, CaMV 35S terminator; Tnos, nopaline synthase terminator; LB, left border; RB, right border. (A) pK7WG2D-GFP (Martinez-Marquez et al., 2015); (B) pJCV52-VvGSTU10 (Martinez-Marquez et al., 2017); (C) pJCV52-VvSTS (Hidalgo et al., 2017); (D) pDGB-VvABCB15; (E) pDGB-VvSTS; (F) pDGB-VvABCB15-YFP.

**REFERENCES**

Bensadoun A, Weinstein D. Assay of proteins in the presence of interfering materials. Anal. Biochem. 1975: 70: 241-250.

Granier F, Van de Walle C. Extraction of plant proteins for two-dimensional electrophoresis. Electrophoresis. 1988: 9: 712-718.

Raghupathi RN, Diwan AM. A protocol for protein estimation that gives a nearly constant color yield with simple proteins and nullifies the effects of four known interfering agents: microestimation of peptide groups. Anal. Biochem. 1994: 219: 356−359.

Silva JC, Gorenstein MV, Li GZ, Vissers JP, Geromanos SJ. Absolute quantification of proteins by LCMSE: a virtue of parallel MS acquisition. Mol Cell Proteomics. 2006: 5(1):144-56.

Vitulo N, Forcato C, Carpinelli EC, Telatin A, Campagna D, D'Angelo M, Zimbello R, Corso M, Vannozzi A, Bonghi C, Lucchin M, Valle G. A deep survey of alternative splicing in grape reveals changes in the splicing machinery related to tissue, stress condition and genotype. BMC Plant Biol. 2014:14:99.

Zhang C, Dai Z, Ferrier T, Orduña L, Santiago A, Peris A, Wong DCJ, Kappel C, Savoi S, Loyola R, Amato A, Kozak B, Li M, Liang A, Carrasco D, Meyer-Regueiro C, Espinoza C, Hilbert G, Figueroa-Balderas R, Cantu D, Arroyo-Garcia R, Arce-Johnson P, Claudel P, Errandonea D, Rodríguez-Concepción M, Duchêne E, Huang SC, Castellarin SD, Tornielli GB, Barrieu F, Matus JT. MYB24 orchestrates terpene and flavonol metabolism as light responses to anthocyanin depletion in variegated grape berries. Plant Cell. 2023 Nov 30;35(12):4238-4265.
