# Supplementary figures and images for: The grapevine ABC transporter B family member 15 is a trans-resveratrol transporter out of grapevine cells
Source: Front Plant Sci. 2025 Jan 20;15:1450638. doi: 10.3389/fpls.2024.1450638 (PMC11792551; doi:10.3389/fpls.2024.1450638)

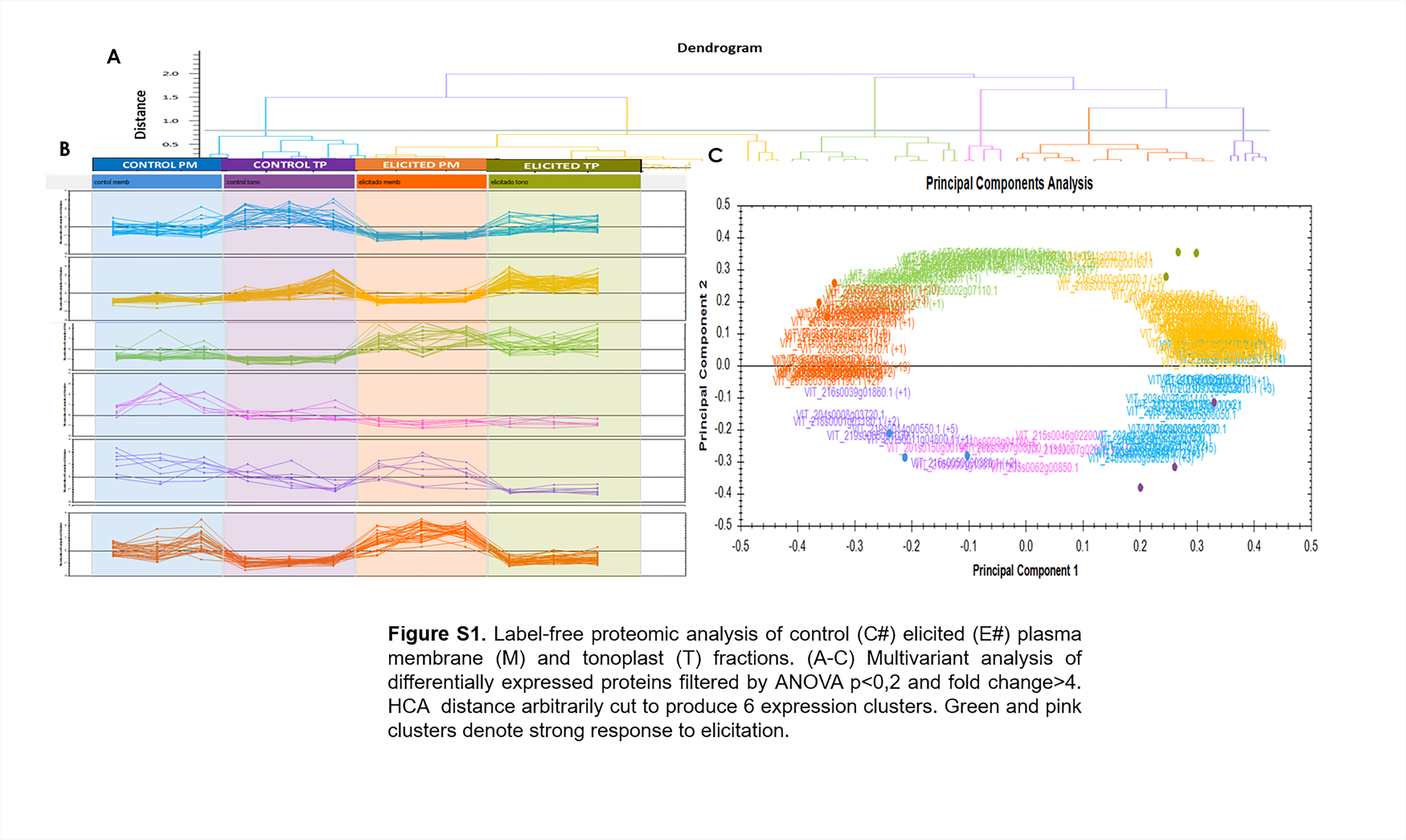

Supplement: Supplementary file 1 [file DataSheet1.zip › Figure S1.tif]

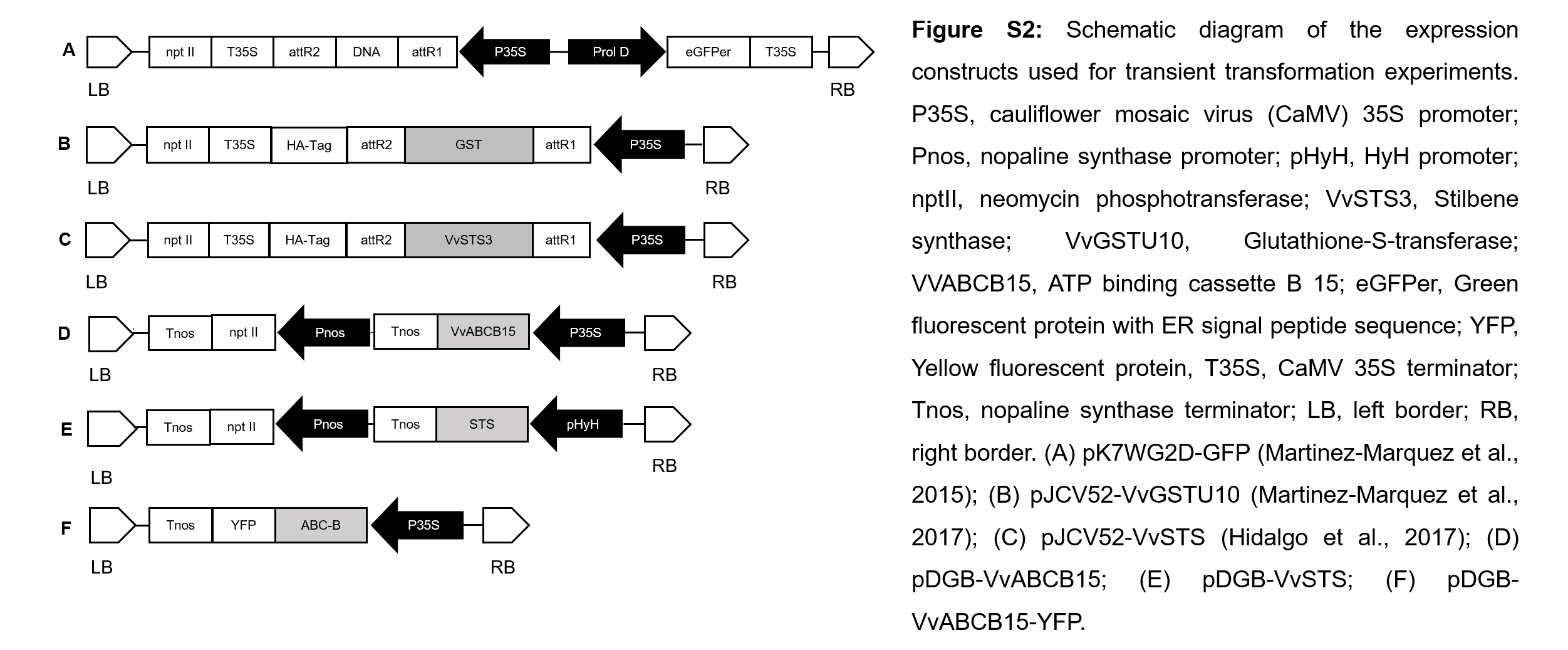

Supplement: Supplementary file 1 [file DataSheet1.zip › Figure S2.tif]

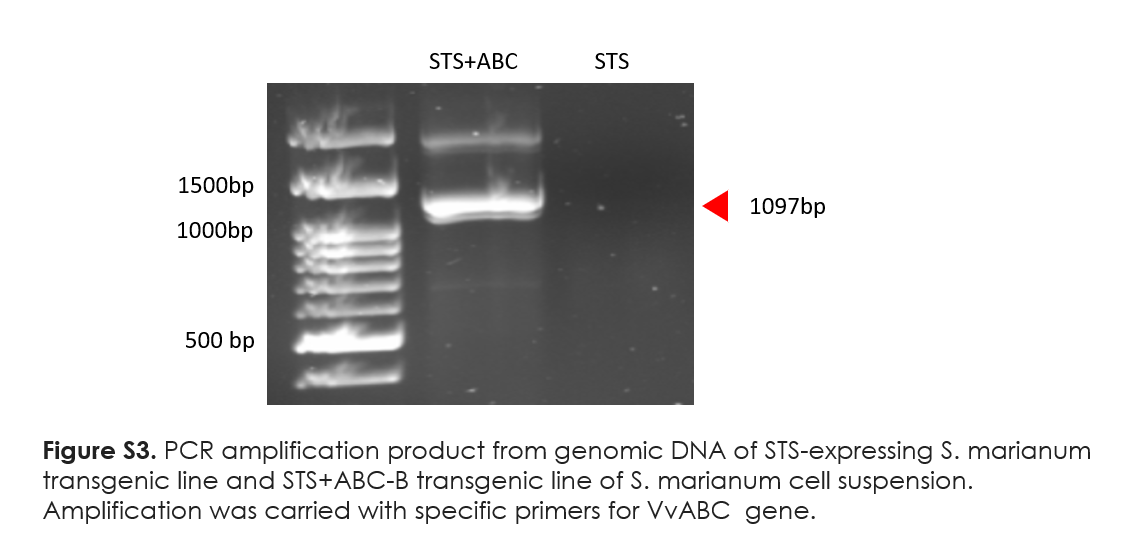

Supplement: Supplementary file 1 [file DataSheet1.zip › Figure S3.tif]

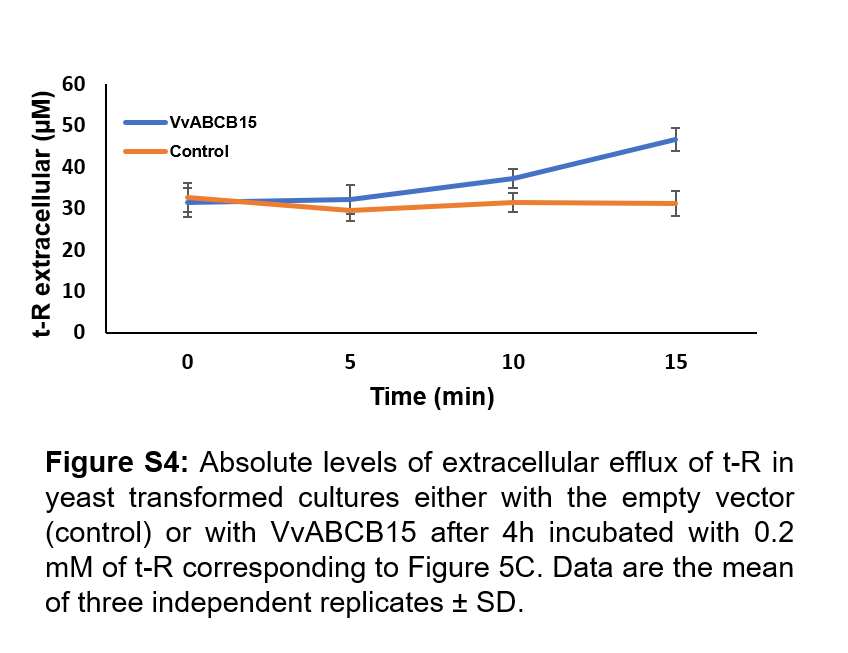

Supplement: Supplementary file 1 [file DataSheet1.zip › Figure S4.tif]

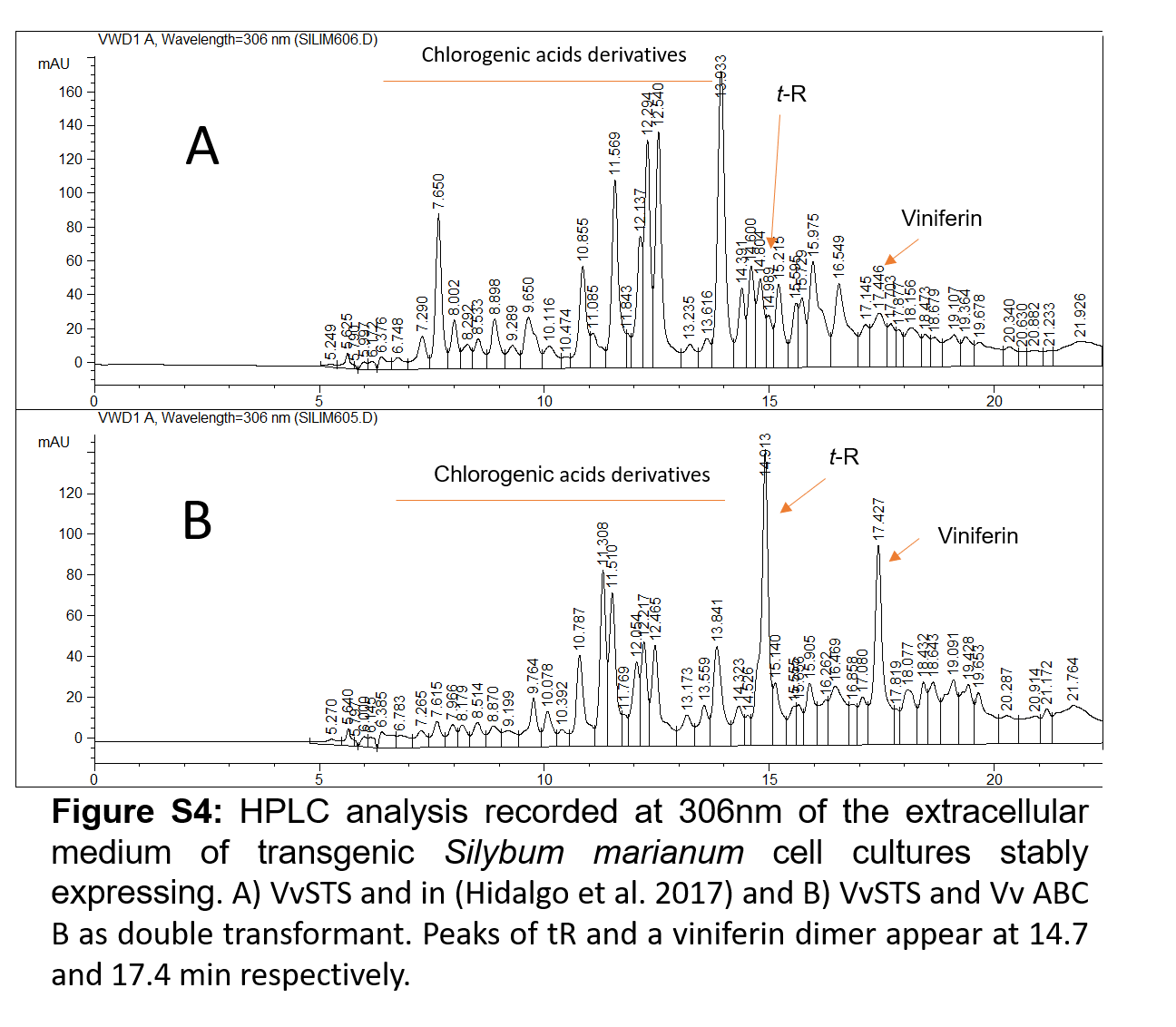

Supplement: Supplementary file 1 [file DataSheet1.zip › Figure S5.tif]
